# The extracellular loop of the membrane permease VraG interacts with GraS to sense cationic antimicrobial peptides in *Staphylococcus aureus*

**DOI:** 10.1371/journal.ppat.1009338

**Published:** 2021-03-01

**Authors:** Junho Cho, Stephen K. Costa, Rachel M. Wierzbicki, William F. C. Rigby, Ambrose L. Cheung

**Affiliations:** 1 Department of Microbiology and Immunology, Geisel School of Medicine, Dartmouth College, Hanover, New Hampshire, United States of America; 2 Department of Medicine, Geisel School of Medicine, Dartmouth-Hitchcock Medical Center, Lebanon, New Hampshire, United States of America; National Institutes of Health, UNITED STATES

## Abstract

Host defense proteins (HDPs), aka defensins, are a key part of the innate immune system that functions by inserting into the bacterial membranes to form pores to kill invading and colonizing microorganisms. To ensure survival, microorganism such as *S*. *aureus* has developed survival strategies to sense and respond to HDPs. One key strategy in *S*. *aureus* is a two-component system (TCS) called GraRS coupled to an efflux pump that consists of a membrane permease VraG and an ATPase VraF, analogous to the BceRS-BceAB system of *Bacillus subtilis* but with distinct differences. While the 9 negatively charged amino acid extracellular loop of the membrane sensor GraS has been shown to be involved in sensing, the major question is how such a small loop can sense diverse HDPs. Mutation analysis in this study divulged that the *vraG* mutant phenocopied the *graS* mutant with respect to reduced activation of downstream effector *mprF*, reduction in surface positive charge and enhanced 2 hr. killing with LL-37 as compared with the parental MRSA strain JE2. *In silico* analysis revealed VraG contains a single 200-residue extracellular loop (EL) situated between the 7^th^ and 8^th^ transmembrane segments (out of 10). Remarkably, deletion of EL in VraG enhanced *mprF* expression, augmented surface positive charge and improved survival in LL-37 vs. parent JE2. As the EL of VraG is rich in lysine residues (16%), in contrast to a preponderance of negatively charged aspartic acid residues (3 out of 9) in the EL of GraS, we divulged the role of charge interaction by showing that K380 in the EL of VraG is an important residue that likely interacts with GraS to interfere with GraS-mediated signaling. Bacterial two-hybrid analysis also supported the interaction of EL of VraG with the EL of GraS. Collectively, we demonstrated an interesting facet of efflux pumps whereby the membrane permease disrupts HDP signaling by inhibiting GraS sensing that involves charged residues in the EL of VraG.

## Author summary

TCS have been used by bacteria to sense and respond to environmental stimuli. In *S*. *aureus*, the bacterial defense against host HDPs is mounted from GraRS where GraS is the membrane sensor comprising two transmembrane segments framing a small extracellular loop, and a cytoplasmic tail. We have shown previously that the critical sensing moiety for HDPs in *S*. *aureus* is the 9-residue extracellular loop of the sensor GraS that contains 3 negatively charged aspartic acid residues critical to HDP sensing. However, the 9-residue loop is predicted to be too flexible to confer proper conformation for the specificity response to divergent HDPs in *S*. aureus. The genes encoding GraRS lies adjacent to an efflux system encoded by *vraFG* where VraG is the membrane permease and VraF is the ATPase. We showed that VraG contributes to sensing of HDPs mediated by GraS. More specifically, the 200-residue lysine rich extracellular loop of VraG interferes with GraS sensing of HDP via charge interaction mediated in part by K380. This finding represents another interesting facet of efflux pump whereby the membrane permease interferes with HDP sensing by inhibiting GraS signaling.

## Introduction

Methicillin-resistant *staphylococcus aureus* (MRSA) is the causative agent for a myriad of serious human infections, ranging from septicemia, endocarditis, osteomyelitis, pneumonia, septic arthritis and cellulitis [1, 2]. Although antibiotics have been used to treat these infections, these treatments often led to evolution of more resistant MRSA clones including vancomycin-intermediate MRSA (VISA) [3, 4] and, in some cases, VRSA [5]. Accordingly, there is a great need to identify new targets for therapeutic interventions that do not result in an increase in drug resistance. One overlooked area that shows promise is to augment host defense to kill these resistant bacteria by mobilizing host bactericidal proteins to kill offending pathogens.

Besides anatomic barriers, a crucial element in host defense against *S*. *aureus* infections is innate immunity, which entails alveolar macrophages, polymorphonuclear leukocytes (PMNs), and “host defense peptides” (HDPs) [6]. Found in mammalian neutrophils (PMNs), macrophages and epithelial cells, HDPs (a.k.a. defensins such as hBD-2, hBD-3, LL-37, hNP-1 etc.) represent a family of short but mostly cationic bactericidal peptides that bind the negatively charged microbial surface to form pores in the bacterial membrane to exert lethality [7]. *S*. *aureus* is selectively resistant to HDPs, exhibiting resistance to human beta-defensin (hBD-2) but moderately sensitive to LL-37 (PMNs and epithelial cells), hNP-1 (PMN) and hBD-3 (keratinocytes) [8–13].

One avenue for bacterial response to host stimuli is mediated by two-component regulatory systems (TCS), known for their ability to sense and respond to external signal(s). Gra(X)RS, also called APS which stands for antimicrobial peptide system [14], is one particular TCS system in *S*. *aureus* that senses and responds to bactericidal HDPs [11, 14–17]. Upon sensing selective HDPs, the histidine kinase (HK) GraS becomes auto phosphorylated, followed by phosphorelay onto the response regulator GraR. Phosphorylation of GraS was thought to be facilitated by GraX [11]. Phosphorylated GraR then induces expression of *mprF* and *dltABCD*, resulting in membrane phospholipid lysylation and teichoic acid alanylation, respectively. These post-translational modifications result in an increase in relative cell surface positive charge to dissuade interaction between *S*. *aureus* and cationic HDPs, culminating in HDP resistance [13].

Among histidine kinases in bacteria, GraS belongs to a unique subset (~ 150 genomes) called Intramembrane histidine kinase (IM-HK), with each HK comprising two transmembrane helices framing a very short extracellular loop (EL), often <10 residues, for sensing [15, 17]. An important question is how GraS, as a member of IM-HK, senses selective HDPs with its short 9-residue EL. Mutagenesis studies by Li *et al*. [14] and our group [15] suggest that the EL of GraS is the putative sensor for HDP. Incidentally, a large group of IM-HKs including GraS has been found to have a topological link to adjacent ABC transporter genes [16, 18]. We were interested to determine if these adjacent genes contribute to GraS-mediated sensing since data from *B*. *subtilis* suggest a role for the membrane permease in sensing (e.g. sensing bacitracin by BceAB where BceB is the permease) [19, 20].

Located directly downstream of *graRS*, *vraG* encodes a membrane permease while *vraF* codes for an ATPase to provide energy for efflux. While GraRS regulates the expression of *vraFG* [16], surprisingly, our data here showed that the *vraG* mutant of JE2 phenocopied the *graS* mutant with respect to HDP sensing. Thus, VraG, more than an efflux pump, facilitates GraS-mediated sensing of HDPs. Structural prediction revealed that VraG comprises 10 transmembrane domains, with a single 200-residue extracellular loop (EL) situated between the 7^th^ and 8^th^ transmembrane segments. We found that VraG, devoid of the EL in MRSA strain JE2, exhibited enhanced sensing to cationic peptides vs. the parental strain, with increasing activation of *mprF*, reducing binding of the cationic dye cytochrome c and improving survival upon 2 hr. exposure to LL-37 while deletion of *vraG* led to diametrically opposed phenotypes. Curiously, 16% of the residues in the EL of VraG are positively charged lysine residues, with stretches of lysines in some regions. Recognizing that 33% of the 9-residue EL sensing domain of GraS are negatively charged aspartic acid residues, we showed that one lysine residue, K380, in the EL of VraG plays a pivotal role in repressing GraS sensor activity for cationic peptides. Bacterial two-hybrid analysis (BACTH) also confirmed the importance of K380 in the EL of VraG in its interaction with GraS. Conversely, GraS protein with a D-35-37-41K mutation in the EL also exhibited reduced interaction with native VraG vs. the native GraS protein control. Importantly, addition of soluble native EL peptide of VraG or the K388A EL peptide, but not the K380A EL peptide, to a ΔEL *vraG* mutant reduced survival of the ΔEL *vraG* mutant in a 2-hr. killing assay with LL-37. These results are consistent with the masking effect of EL of VraG to reduce GraS-activation to improve survival upon encountering HDP such as LL-37. Based on these data, we postulate that specific lysine residue(s) on the EL of VraG likely reduces cognate sensing of HDPs or cationic peptides by the 9-reside EL of GraS, presumably via charge interaction. Collectively, these data provide new insights into role of EL of membrane permease in sensing by modulating GraS-mediated sensing of HDPs in *S*. *aureus*. We also believe that this knowledge on sensing and response to HDP is an important area of *S aureus* pathogenesis that will help facilitate development of strategies to subvert these pivotal mechanisms (e.g., block sensing with small molecules or peptides) to augment bacterial clearance by the host innate immune system.

## Results

### Deletion of *vraG* phenocopied the *graS* mutant of JE2

Based on molecular modeling [21, 22], VraG is predicted to be a membrane protein bearing the core structure of an ABC transporter (i.e. membrane permease) with 10 transmembrane segments and one large 200-residue EL between the 7^th^ and 8^th^ transmembrane segments (S1 Fig). Previous studies have largely focused on the regulation of *vraFG* by the TCS GraRS [16] where the primary sensor domain of GraS appears to reside in the 9-residue EL [11, 16]. Replacement of the EL of VraG in *S*. *aureus* [11, 23] with its counterpart from VraE, a membrane permease responsible for bacitracin resistance, led to enhanced bacitracin resistance while increasing sensitivity to colistin, a proxy to polymyxin B (PMB). This finding supports the notion that VraG may be involved in the signal transduction of GraRS upon exposure to HDPs or cationic peptides [11].

To validate the effects of VraG on sensing of HDPs, we constructed a Δ*vraG* mutant of JE2, a derivative of MRSA USA300 [24], followed by chromosomal complementation (Table 1). The Δ*graS* mutant and its complemented strain were used as respective negative and positive controls for HDP sensing. We first measured the overall sensitivity of these mutants to PMB, complying with the CLSI standard [25]. As seen in Table 2, the Δ*vraG* mutant displayed increased susceptibility to PMB by ~16 folds, akin to what has been found with the Δ*graS* strain as compared to the parent strain JE2.

**Table 1:** Strains and plasmids.

Strains and plasmids	Features	Reference
JE2	MRSA (a derivative of USA300 LAC)	[24]
IM08B	An *E*. *coli* recipient that serves as an intermediate for proper plasmid methylation prior to *S*. *aureus* transformation	[26]
DHT1	An adenylate cyclase (*cya*) deficient derivate of DH1, BACTH strain; Tet^r^	[27]
BL21(DE3)	An *E*. *coli* used for protein expression controlled by T7 promoter	[28]
C43(DE3)	An *E*. *coli*, a derivative of BL21(DE3), used for toxic or membrane protein expression	[29]
pMAD	A temperature sensitive shuttle plasmid for introducing mutations into *S*. *aureus*; Erm^r^ for *S*. *aureus*, Amp^r^ for *E*. *coli*.	[30]
pALC1484	A plasmid harboring promoterless GFPuvr; Cm^r^ for *S*. *aureus*, Amp^r^ for *E*. *coli*.	[31]
pKT25	A plasmid expressing catalytic N-terminal domain, T25 fragment, of adenylate cyclase in *Bordetella pertussis*; Kan^r^	[32]
pUT18	A plasmid expressing catalytic C-terminal domain, T18 fragment, of adenylate cyclase in *Bordetella pertussis*; Amp^r^	[32]
pET14b TEV	pET14b plasmid in which thrombin cleavage site is replaced by TEV cleavage site	This study
JE2 Δ*graS*	JE2 with *graS* deletion	This study
JE2 Δ*vraG*	JE2 with *vraG* deletion	This study
JE2 ΔEL of *vraG*	JE2 with extracellular loop of *vraG* deleted	This study
JE2 *vraG* mutant 1	JE2 with *vraG* K327A, K331A and K343A	This study
JE2 *vraG* mutant 2	JE2 with *vraG* K351A, K357A, K360A, K367A and K369A	This study
JE2 *vraG* mutant 3	JE2 with *vraG* K380A and K388A	This study
JE2 *vraG* mutant 4	JE2 with *vraG* K402A, K406A, K408A, K409A, K412A, K418A and K419A	This study
JE2 *vraG* mutant 5	JE2 with *vraG* K425A, K427A and K432A	This study
JE2 *vraG* mutant 6	JE2 with *vraG* K451A, K458A, K461 and K463A	This study
JE2 *vraG* mutant 7	JE2 with *vraG* K474A, K476A, K477A, K485A, K486A, K488A, and K491	This study
JE2 *vraG* K380A	JE2 with *vraG* K380A mutation	This study
JE2 *vraG* K388A	JE2 with *vraG* K388A mutation	This study
JE2 Δ*graS* complement	JE2 Δ*graS* complemented with native *graS*	This study
JE2 Δ*vraG* complement	JE2 Δ*vraG* complemented with native *vraG*	This study
JE2 *vraG* K380A complement	JE2 *vraG* K380A mutant replaced by native *vraG*	This study
IM08B pALC1484::*mprF* promoter	IM08B with pALC1484 harboring *mprF* promoter	This study
IM08B pALC1484::*dltA* promoter	IM08B with pALC1484 harboring *dltA* promoter	This study
JE2 pALC1484::*mprF* promoter	JE2 with pALC1484::*mprF* promoter	This study
JE2 Δ*vraG* pALC1484::*mprF* promoter	JE2 Δ*vraG* with pALC1484::*mprF* promoter	This study
JE2 ΔEL of *vraG* pALC1484::*mprF* promoter	JE2 ΔEL of *vraG* with pALC1484::*mprF* promoter	This study
JE2 *vraG* mutant 1 pALC1484::*mprF* promoter	JE2 *vraG* mutant 1 with pALC1484::*mprF* promoter	This study
JE2 *vraG* mutant 2 pALC1484::*mprF* promoter	JE2 *vraG* mutant 2 with pALC1484::*mprF* promoter	This study
JE2 *vraG* mutant 3 pALC1484::*mprF* promoter	JE2 *vraG* mutant 3 with pALC1484::*mprF* promoter	This study
JE2 *vraG* mutant 4 pALC1484::*mprF* promoter	JE2 *vraG* mutant 4 with pALC1484::*mprF* promoter	This study
JE2 *vraG* mutant 5 pALC1484::*mprF* promoter	JE2 *vraG* mutant 5 with pALC1484::*mprF* promoter	This study
JE2 *vraG* mutant 6 pALC1484::*mprF* promoter	JE2 *vraG* mutant 6 with pALC1484::*mprF* promoter	This study
JE2 *vraG* mutant 7 pALC1484::*mprF* promoter	JE2 *vraG* mutant 7 with pALC1484::*mprF* promoter	This study
JE2 *vraG* K380A pALC1484::*mprF* promoter	JE2 *vraG* K380A with pALC1484::*mprF* promoter	This study
JE2 *vraG* K388A pALC1484::*mprF* promoter	JE2 *vraG* K388A with pALC1484::*mprF* promoter	This study
JE2 Δ*vraG* complement pALC1484::*mprF* promoter	JE2 Δ*vraG* complement with pALC1484::*mprF* promoter	This study
JE2 ΔEL of *vraG* complement pALC1484::*mprF* promoter	JE2 ΔEL of *vraG* complement with pALC1484::*mprF* promoter	This study
JE2 *vraG* K380A complement pALC1484::*mprF* promoter	JE2 *vraG* K380A complement with pALC1484::*mprF* promoter	This study
DHT1 pKT25 pUT18	DHT1 harboring two empty plasmids	-
DHT1 pKT25::*vraG* pUT18::*graS*	DHT1 harboring two plasmids expressing VraG with T25 and GraS with T18	[11]
DHT1 pKT25::ΔEL *vraG* pUT18::*graS*	DHT1 harboring two plasmids expressing ΔEL VraG with T25 and GraS with T18	This study
DHT1 pKT25::*vraG* mutant 3 pUT18::*graS*	DHT1 harboring two plasmids expressing VraG mutant 3 with T25 and GraS with T18	This study
DHT1 pKT25::*vraG* K380A pUT18::*graS*	DHT1 harboring two plasmids expressing VraG K380A with T25 and GraS with T18	This study
DHT1 pKT25::*vraG* K388A pUT18::*graS*	DHT1 harboring two plasmids expressing VraG K388A with T25 and GraS with T18	This study
DHT1 pKT25::*vraG* pUT18::*graS* D35, 37, 41K	DHT1 harboring two plasmids expressing VraG with T25 and GraS D35, 37, 41K with T18	This study
DHT1 pKT25::*vraG* pUT18::*graS* D35K	DHT1 harboring two plasmids expressing VraG with T25 and GraS D35K with T18	This study
DHT1 pKT25::*vraG* pUT18::*graS* D37K	DHT1 harboring two plasmids expressing VraG with T25 and GraS D37K with T18	This study
DHT1 pKT25::*vraG* pUT18::*graS* D41K	DHT1 harboring two plasmids expressing VraG with T25 and GraS D41K with T18	This study
JE2 *vraG* with HA	JE2 expressing HA tag on C-terminal of *vraG*	This study
JE2 ΔEL *vraG* with HA	JE2 expressing HA tag on C-terminal of ΔEL *vraG*	This study
JE2 *vraG* mutant 3 with HA	JE2 expressing HA tag on C-terminal of v*raG* mutant 3	This study
JE2 *vraG* K380A with HA	JE2 expressing HA tag on C-terminal of *vraG* K380A	This study
JE2 *vraG* K388A with HA	JE2 expressing HA tag on C-terminal of *vraG* K388A	This study
BL21(DE3) pET14b TEV::EL *vraG*	An *E*. *coli* expressing EL of VraG	This study
BL21(DE3) pET14b TEV::EL *vraG* mutant 3	An *E*. *coli* expressing EL of VraG mutant 3	This study
BL21(DE3) pET14b TEV::EL *vraG* K380A	An *E*. *coli* expressing EL of VraG K380A	This study
BL21(DE3) pET14b TEV::EL *vraG* K388A	An *E*. *coli* expressing EL of VraG K388A	This study
C43(DE3) pET14b TEV::EL *vraE*	An *E*. *coli* expressing EL of VraE	This study

**Table 2:** MICs of PMB for JE2 mutants.

Strains	MICs of PMB (ug/ml)
JE2	128–256
JE2 Δ*graS*	4–16
JE2 Δ*vraG*	8–16
JE2 ΔEL of *vraG*	16–32
JE2 *vraG* mutant 1*	16–32
JE2 *vraG* mutant 2*	16–32
JE2 *vraG* mutant 3*	32–64
JE2 *vraG* mutant 4*	16–32
JE2 *vraG* mutant 5*	16–32
JE2 *vraG* mutant 6*	16–32
JE2 *vraG* mutant 7*	16–32
JE2 *vraG* K380A	32–64
JE2 *vraG* K388A	64–128
JE2 Δ*graS* complement	128–256
JE2 Δ*vraG* complement	64–128
JE2 ΔEL of *vraG* complement	64–128
JE2 *vraG* K380A complement	64–128

Overnight cultures were diluted and plated with various concentrations of PMB, following CLSI guideline. The MIC values were obtained from at least three biological replicates. The ΔEL of *vraG* represents a 180-residue deletion of the extracellular loop of *vraG*, leaving 10 residues on each end. The complemented strains were constructed by homologous recombination to return the respective mutated genes to the native configuration.

*JE2 *vraG* mutant 1: K327, 331 and 343A; mutant 2: K351, K357, K360, K367 and K369A; mutant 3: K380 and K388A; mutant 4: K402, 406, 408, 409, 412, 418 and 419A; mutant 5: K425, K428 and 432A; mutant 6: K451, 458, 461 and 463A; mutant 7: K474, 476, 477, 484, 486, 488 and 491A.

**Fig 1:**
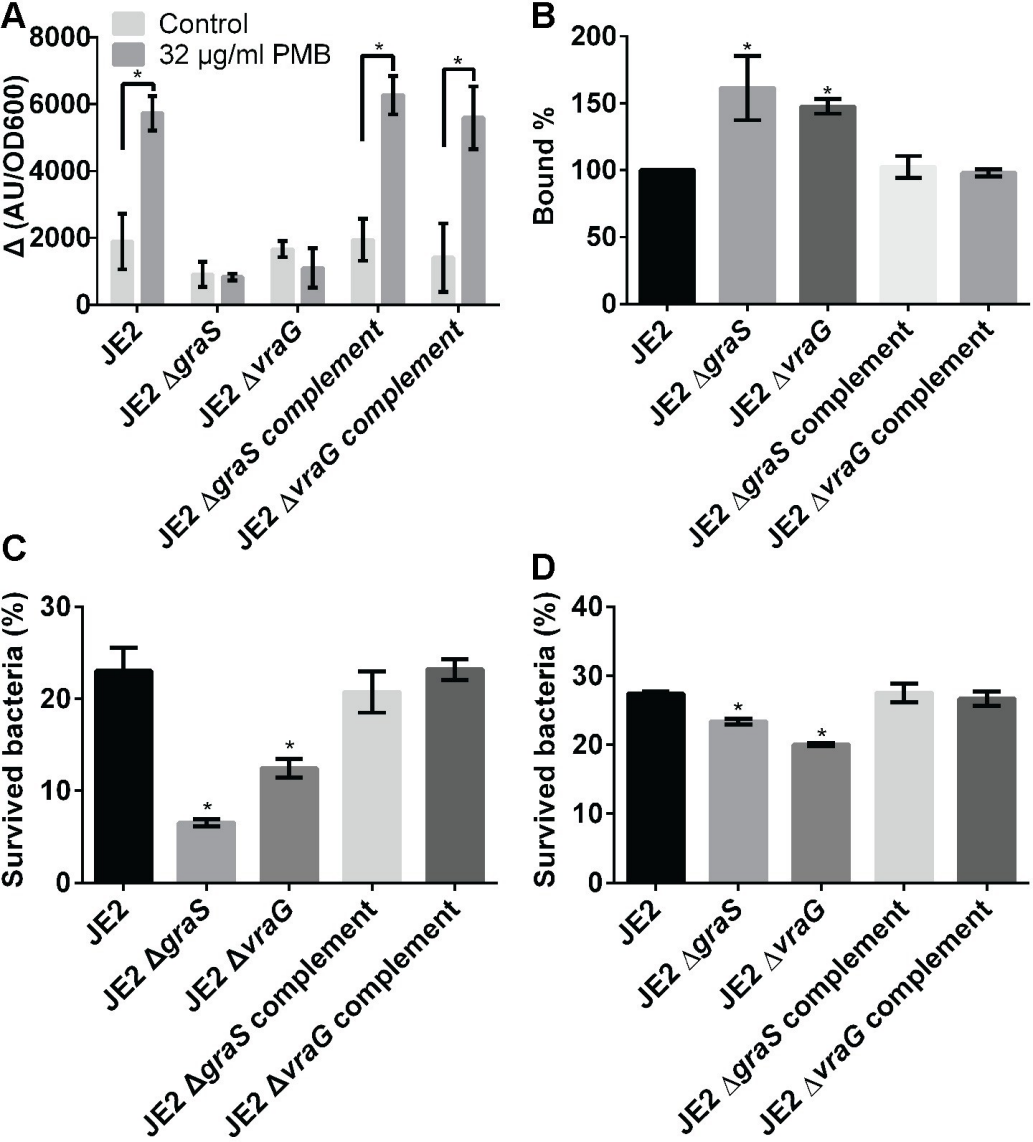
Δ*vraG* mutant phenocopies the Δ*graS* mutant. **(A)** The expression of the *mprF* promoter driving GFPuvr fusion upon PMB treatment. Strains with *mprF*-promoter fused to the GFPuvr reporter grown to mid-log phase were treated with 32 μg/ml PMB or water (control) and incubated for additional 30 min at 37°C. The OD600s and A.U. (a fixed gain value at 120) of the strains were measured at two time points (before and 30 min after the addition of PMB). The means (the heights of bars) and SDs (error bars) were determined from three biological replicates. * represents p < 0.05 between two samples using Student-t test. **(B)** Cytochrome c binding assay. Cells in mid-log phase were resuspended in 0.25 mg/ml cytochrome c solution and incubated for 10 min at room temperature. The binding values of cytochrome c were determined by subtracting unbound amount of cytochrome c in supernatants after pelleting. **(C)** LL-37 susceptibility assay. Cells exposed to 2.5 μg/ml of LL-37 were incubated for 2 hr. at 37°C and percent survival was calculated, with the inoculum set at 100%. **(D)** PMN assay. Opsonized cells in mid-log phase were incubated with PMN for 1 hr. at 37°C. The bar graphs are depicted as means (the heights of bars) with standard deviations (SDs, error bars) calculated from three biological replicates. For the susceptibility assays (LL-37 and PMN assay), the survival rates of bacteria were calculated from ratios of initial and survived number of cells. The results for susceptibility assays are representative of three independent experiments. * indicates p < 0.05 against the parent strain in **B, C** and **D**. Student-t test were used to compare two samples evaluated at the same time.

To confirm if increased sensitivity of the Δ*vraG* mutant to PMB is associated with GraRS, we constructed in pALC1484 derivatives of the GPFuvr reporter, which carries a S65T mutation in the *gfp*_*uv*_ gene (Clontech) to enable a shift in emission spectra from UV to Ex/Em = 488/507_nm_ [31, 33], fused to promoters of *mprF* or *dltA*, which are downstream targets of the GraRS regulon [34]. For our purpose, we utilized data with the *mprF* promoter, but similar findings were obtained with the *dltA* promoter fusion with reporter, albeit at a much larger magnitude. In agreement with the PMB MICs, the Δ*vraG* and Δ*graS* mutants exhibited significantly reduced *mprF* expression under induction with 32 μg/ml PMB as well as a lower constitutive level of *mprF* expression, corresponding to reduced level of fluorescence, compared to the parent strain. (Fig 1A). Lower concentrations of PMB (1, 2, 4 and 8 μg/ml) also failed to induce *mprF* expression in the Δ*vraG* mutant. In contrast, the complemented strains were able to exhibit PMB-mediated induction similar to the parental strain JE2.

If VraG indeed contributes to GraS-mediated sensing of HDPs, we expect induction of *mprF* and *dltABCD* by GraS to result in an increase in surface positive charge due to lysylation and D-alanylation of membrane phospholipid and cell-wall teichoic acid, respectively. Indeed, the *ΔvraG* and Δ*graS* mutants appeared to bind more cytochrome c, a positively charged dye, correlating with reduced cell surface positive charge (Fig 1B). A consequence of a reduction in surface positive charge is to facilitate interaction with positively charged HDPs, leading to decreased survival or increased sensitivity to specific HDPs within the induction window (~30 min to 2 hr.). Accordingly, we performed the 2-hr. survival assay with LL-37, an HDP present on the skin and in PMNs (Fig 1C), as a reflection of the ability of VraG to mediate GraS-mediated sensing of HDPs. As anticipated, the Δ*graS* mutant was highly susceptible to LL-37, with less than 10% of the cells surviving while survival of the parent JE2 stood at over 20%. Although the Δ*vraG* mutant survived slightly better (>10%) than the isogenic Δ*graS* mutant, it remained highly susceptible to LL-37 vs. the parent JE2. As PMNs are noted to contain LL-37, we conducted the PMN survival assay, showing that both Δ*graS* and Δ*vraG* mutants displayed statistically significant reduction in bacterial survivability upon exposure to PMNs, but restored to parental level upon complementation (Fig 1D). Collectively, these data indicated that deletion of *vraG* reduces bacterial resistance to HDPs by impairing GraS-mediated signaling of downstream effectors (e.g., *mprF*) to alter the surface positive charge. We also found that the VraG effect is likely mediated via GraS due to the following: 1) *vraG* mutant generally phenocopies the *graS* mutant (as described above); 2) exogenous activation of the *vraFG* promoter in a *graS* mutant with PMB did not augment the *mprF* promoter.

### The EL of *vraG* reduces activation of the TCS sensor GraS

Bacterial two hybrid analysis by Falord *et al*. [11] as well as data from our group (see bacterial 2-hybrid analysis below) reveal a direct interaction between VraG and GraS. Based on mutation analysis, we previously demonstrated that the three negatively-charged aspartic residues on the 9-residue EL of GraS are responsible for HDP sensing [15, 35]. However, the short EL of GraS is predicted to be extremely flexible and hence lacks conformation to help explain the structural basis of HDP specificity in *S*. *aureus* (e.g., resistant to h-BD2 but sensitive to LL-37). Importantly, the EL of VraG has been shown by Hiron *et al*. to be an important component in HDP signaling since replacement of native EL with that from VraE, a permease for bacitracin resistance [23], led to bacitracin resistance. Given that the Δ*vraG* mutant phenotypically resembles the Δ*graS* mutant, we surmised that the EL of VraG may contribute to GraS-mediated sensing of HDPs by modulating the sensing ability of GraS.

**Fig 2:**
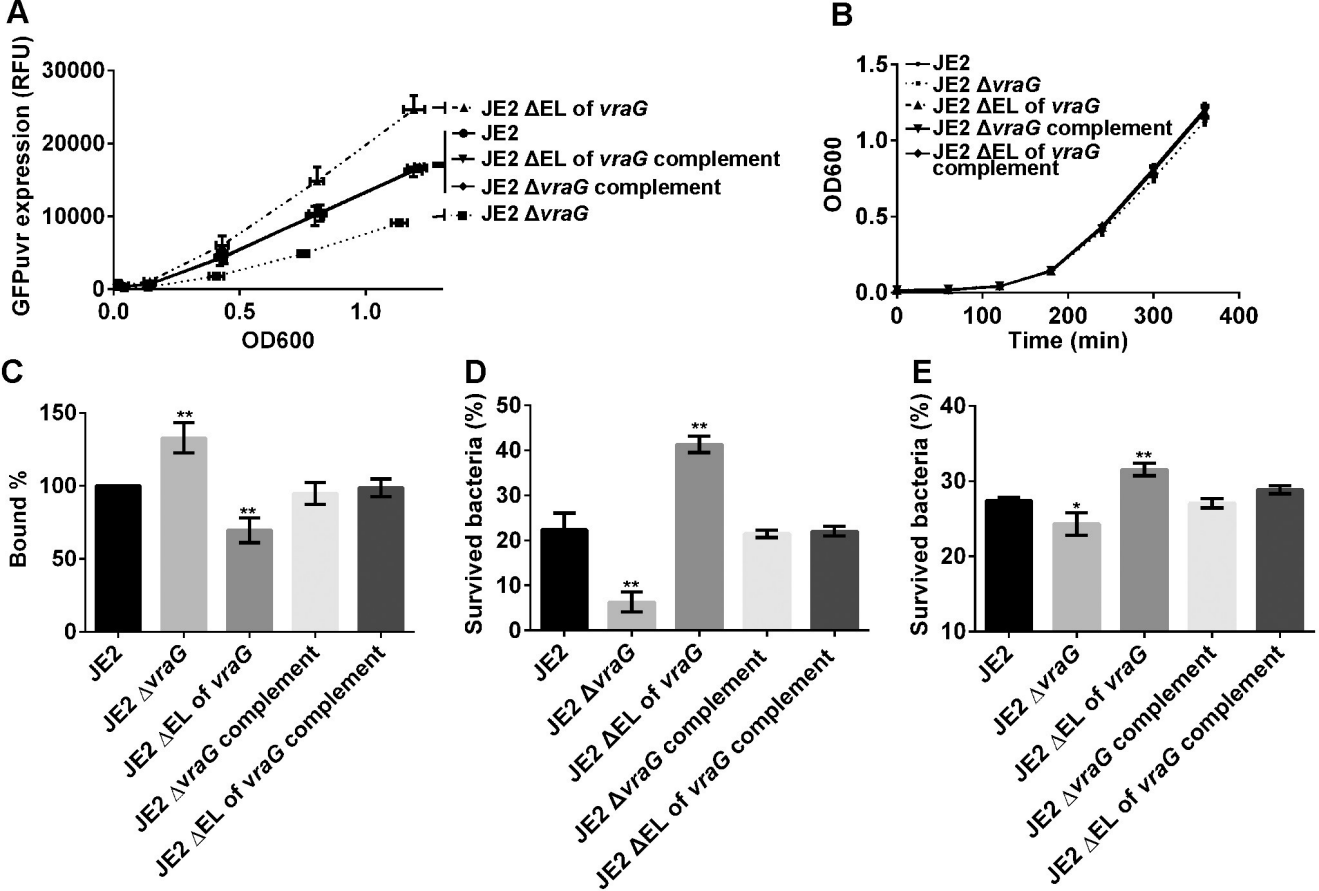
The ΔEL *vraG* mutant enhances GraS-mediated signaling of HDP. **(A)** The *mprF* promoter fusion with GFPuvr reporter assay. **(B)** Cell growth of assorted *vraG* mutants and the parent JE2. Fluorescent intensities and OD600 were measured every hour for 6 hours. The data were plotted by fluorescence arbitrary unit (A.U.) with fixed gain at 145 vs. OD_600_ with error bars (horizontal: SDs of A.U., vertical: SDs of OD_600_). The growth curves of the depicted strains were given as OD_600_ vs. Time (min) with error bars (SDs). The data were obtained from three independent experiments. **(C)** Cytochrome c binding assay, **(D)** LL-37 susceptibility assay, **(E)** PMN assay were performed as previously described. The asterisks * and ** for **C**, **D** and **E** indicate p <0.05 and <0.01, respectively, using Student-t test.

For these studies, we constructed a mutant lacking 180 residues of EL (ΔEL *vraG* mutant), leaving 10 EL residues each on both sides to maintain the integrity of the membrane portion of VraG (S1 Fig). The newly constructed ΔEL mutant was transformed with the promoter fusion-GFPuvr plasmid to ascertain *mprF* expression with PMB. Remarkably, the basal level of *mprF* expression in the ΔEL *vraG* mutant was higher than the parent JE2 without induction (S2 Fig) while that of the Δ*vraG* mutant was lower than the parent (Fig 2A). In contrast, the MIC of the ΔEL *vraG* mutant to PMB was 2-4-fold higher than the Δ*vraG* mutant, but lower than the parent (Table 2). Despite elevated *mprF* expression at basal level, expression in the ΔEL *vraG* mutant was not markedly induced with PMB (S2 Fig), possibly due to high basal level of expression; in contrast, the Δ*vraG* mutant exhibited low and non-inducible *mprF* expression, akin to the *graS* mutant (Fig 1A). To confirm elevated *mprF* expression in the ΔEL *vraG* mutant, *mprF* expression profile and growth at OD_600_ were measured at one-hour intervals (Fig 2A and 2B). While cell growth was not affected by the EL deletion, the ΔEL *vraG* mutant displayed enhanced *mprF* expression ~1.5 times the parental and three times the Δ*vraG* mutant levels (the slope values for 95% confidence intervals in linear regression, JE2: 12689–14494; JE2 Δ*vraG* complement: 12587–14554; JE2 ΔEL of *vraG*: 18605–21973; JE2 Δ*vraG*: 6652–7867). Altogether, our results so far implied that the EL of VraG modulates *mprF* expression, likely by impeding signal transduction of the kinase sensor GraS which has been shown previously to respond to PMB by up-regulating *mprF* expression [15, 33, 34].

To determine if increased *mprF* expression in the ΔEL *vraG* mutant led to changes in cell surface positive charge as predicted, we analyzed the ratios of positively charged cytochrome c binding to the ΔEL *vraG* mutant vs. the parent (set at 100%) and Δ*vraG* mutant (Fig 2C). As expected, the ΔEL *vraG* mutant bound less cytochrome c (~20%) than the parent JE2 and complemented mutants while the Δ*vraG* mutant was found to bind more cytochrome c. These results are consistent with increased surface positive charge due to augmented *mprF* expression by GraS activation in the ΔEL *vraG* mutant. We surmise that an increase in surface positive charge would enhance survival upon 2 hr. exposure to LL-37 *in vitro*. That was indeed the case with the ΔEL *vraG* mutant (~40% survival) as compared with the parental strain (~20%) while the survival of the Δ*vraG* mutant was less (<10%) (Fig 2D). The ΔEL *vraG* mutant also displayed enhanced ability to survive in human PMN (containing LL-37) vs. parent JE2 and complemented mutant while the Δ*vraG* mutant survived less well than the parent (Fig 2E). These findings indicate that the EL of VraG likely modulates the effect of HDPs in human PMNs by altering the GraS signaling pathway. In the absence of EL in VraG, the basal GraS signal transduction is elevated, even without induction by cationic peptides (e.g., LL-37 or PMB) while deletion of VraG completely abolishes the GraS-mediated signal, akin to what has been observed in a *graS* mutant.

### Lysine residues in the EL of VraG affect HDP resistance

We have shown that deletion of EL in *vraG* induced *mprF* over-expression and enhanced cell surface positive charge to reduce PMB and LL-37 interaction. This led us to surmise if specific residue(s) in EL of VraG serves to restrict signaling of GraS and hence diminish activation of downstream target genes (e.g., *mprF*). Based on structural analysis, four sections of EL of VraG (Q342-Y372, V396-G416, K428-R457 and Y459-A481) were replaced with inverted sequence of the native gene. However, each of these mutants revealed MIC to PMB and cytochrome c binding to be identical to the Δ*vraG* mutant, indicating that the resultant VraG had been rendered malfunctional.

**Fig 3:**
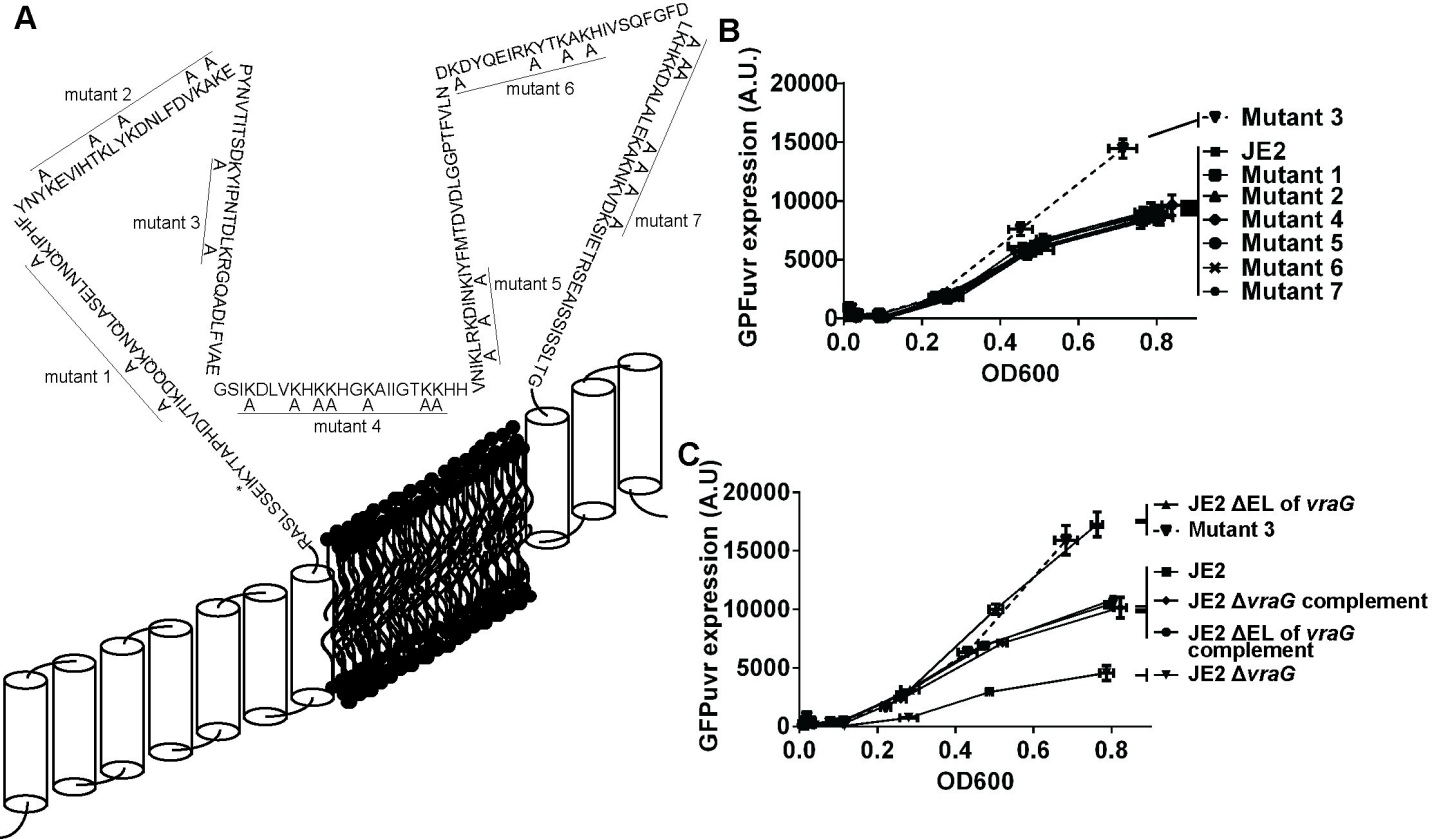
Lysine residue in the EL of VraG is involved in GraS-mediated signaling. **(A)** Pictorial representation of the VraG membrane structure. VraG is consisted of 10 transmembrane segments, 5 extracellular and 4 intracellular loops. There is one large extracellular loop of ~ 200 amino acids harboring 32 lysine residues while the other extracellular loop is small (7–23 residues). Based on the distribution pattern (densely grouped) of lysine residues in the loop, we divided the EL into seven groups and mutated all the lysines in each group to alanine. They were labeled as mutants 1 to 7 (Table 2). The K317 residue of VraG (marked as asterisk) was not included in the mutagenesis plan as this particular residue was close to a transmembrane domain. **(B)**, **(C)** The expression of *mprF* promoter fused with a GFPuvr reporter. Seven lysine group mutants (B) as well as ΔEL and Δ*vraG* mutants and their complements (C) with the GFPuvr reporter were monitored by measuring fluorescent intensities (A.U.) and OD_600_ as previously described. The data were displayed by A.U.s (y-axis) and OD600s (x-axis) with SDs (error bars) of corresponding values calculated from three biological replicates.

To reverse the effect of EL without significantly disrupting the VraG function, we explored the role of positively charged residues in the EL since ~16% (32 in 200) of residues in EL of VraG, in contrast to the membrane segment, are lysine residues (S1 Fig) and the 9-residue EL of GraS responsible for sensing HDPs harbors three negatively charged aspartic residues (D35-37-41) [36]. We speculated that the lysine residues of VraG may mask the negatively charged sensing residues of EL in GraS to limit its sensor activity. As some lysine residues were adjacently congregated within some regions of the loop, we were able to sort the lysine residues into seven groups followed by mutating them into alanine residues (designated mutants 1 to 7) (Table 2 and Figs 3A and S1). To locate the functional lysine residues in the EL of VraG for masking signal transduction by the EL of GraS, the MICs of the seven mutants to PMB was first measured, showing six of the mutants with MIC ranging from 16 to 32 μg/ml but mutant 3 appeared to have a consistently higher MIC (32–64 μg/ml), at a level closer to the parent JE2 (128–256 μg/ml) (Table 2).

To further characterize mutant 3 vs. the other mutants, these strains were transformed with plasmid containing the *mprF* promoter linked to the GFPuvr gene and florescence intensities calibrated to cell density were measured over a time course. Like the ΔEL *vraG* mutant, mutant 3 harboring double lysine mutations (K380A and K388A) induced over-expression of GFPuvr (Fig 3B and 3C) without affecting cell growth (S3 Fig). In contrast to mutant 3, the remaining six mutants appeared to express GFPuvr levels similar to the wild type strain JE2. These data indicated that while most of the lysine mutations in the EL of VraG affect MIC moderately, only the mutant 3 containing K380A and K388A mutations has consistently higher MIC to PMB and was the only one to have higher *mprF* expression (Fig 3B and 3C) via the GraS signaling pathway vs. the parent and other mutants.

### Lysine 380 in EL of VraG regulates GraS signal transduction

**Fig 4:**
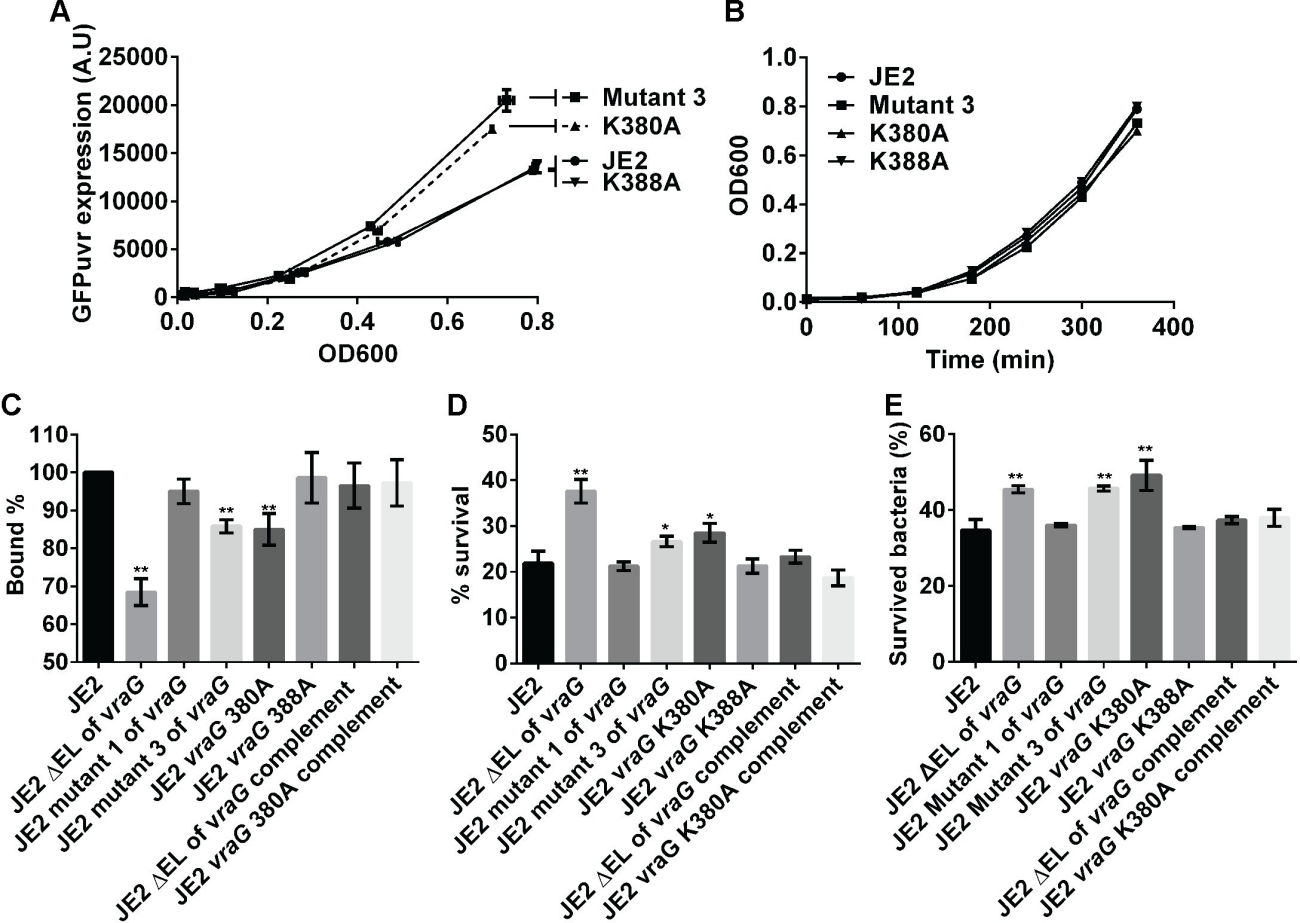
Lysine 380 of VraG interferes with GraS-mediated signaling of HDPs. **(A)** The expression of *mprF* promoter fused to the GPFuvr reporter. **(B)** Cell growth at OD_600_. **(C)** Cytochrome c binding assay. **(D)** 2 hr. LL-37 susceptibility assay. **(E)** PMN assay. These assays were performed, as previously described. Mutant 3 indicates the double mutant (K380, and K388A). Mutant 1 (K327, 331 and 343A) was randomly chosen as a negative control in lysine mutants. The asterisks * and ** for **C**, **D** and **E** indicate p <0.05 and <0.01 vs. the wild type, respectively, using the Student-t tests.

As mutant 3 containing double mutations (K380A and K388A) induced over-expression of *mprF* mirroring that of the ΔEL *vraG* mutant, an effort was undertaken to distinguish the effects of these two lysine residues on GraS signal transduction by mutating individual residues to alanine. Using these mutants, we measured *mprF* expression, using the *mprF* promoter fused to GFPuvr reporter. As shown in Fig 4A, The K380A mutant induced overexpression of GFPuvr, at a level comparable to mutant 3 while the K388A mutant did not. Notably, cell growth was not significantly affected by these two mutations (Fig 4B).

To ascertain if K380 is the active residue that modulates GraS signal transduction, we revisited the cytochrome c binding assay to detect changes in cell surface positive charge in individual mutants. As surface positive charge negatively correlates with the amount of cytochrome c bound, our data suggested that the K380A mutant and mutant 3 harbored a moderate level of (+) charge on the cell surface that was lower than ΔEL *vraG* mutant but still higher than the parent and complemented mutants (Fig 4C). These data on surface positive charge completely mirrored the 2-hr. survival assay with LL-37, showing that the ΔEL *vraG* mutant survived better in LL-37 than the parent JE2 and complemented mutant while mutant 3 and K380A mutant retained intermediate level of survivability in LL-37 (Fig 4D). With a high basal level of *mprF* expression, the ΔEL *vraG* mutant also failed to augment *mprF* expression further upon exposure to PMB but mutant 3 and K380A mutant did (S3 Fig). These results suggest that the ΔEL *vraG* mutant obtained the full potency to enhance GraS signal transduction while the K380 residue in the EL of VraG interferes with the full activation of GraS. The mutant 3 and K380A mutant also had higher survivability in PMN compared to the parent strain, similar to the ΔEL *vraG* mutant (Fig 4E). As mutant 1 (K327, 331 and 343A) and K388A mutant did not differ significantly from each other in cytochrome c binding, LL-37 2 hr. killing assay, and PMN assays, it is likely that these lysine residues within the EL of VraG are not necessary for the sensing function for GraS; however, a combination of these mutations or specific conformational requirement in signaling cannot be entirely ruled out.

Of concern in these assays is whether the mutant EL fragments were expressed as part of the VraG protein or as degraded proteins. To ascertain this, we cloned a HA-tag to the C-terminus of all mutated VraG proteins in MRSA JE2. Cells were lysed and the membrane fractions harvested by ultracentrifugation. Using equivalent amounts of membrane proteins, the samples were resolved in SDS gels (S5 Fig) and blotted onto PVDF membrane. After blocking with 5% skim milk, the membrane was incubated with chicken anti-HA antibody (1:1000) followed by goat anti-chicken antibody (1:5000) conjugated to alkaline phosphatase and developing substrate. As seen in S5 Fig, all versions of mutated proteins including mutant 3, K380A and K388A mutants were expressed at the same molecular weight as the wild type HA-tagged VraG protein whereas VraG with an EL truncation was detected at a lower molecular size. In addition, the untagged VraG protein in JE2 did not react with the anti-HA antibody. Densitometric analysis (S5 Fig legend) disclosed that the VraG band in K380A and ΔEL *vraG* mutants are slightly lower than the wild type, however, it did not follow any specific pattern as the VraG band of mutant 3 harboring K380A and K388A mutations had the most intense band.

Based on expression and phenotypic data, we conclude that K380 in EL of VraG specifically regulates the activation of GraS-mediating signaling, likely by interacting with negatively charged residues(s) on the EL of GraS to interfere with the sensor function. As mutant 3 and K380A mutant differ from the ΔEL *vraG* mutant in terms of lesser overall surface positive charge (i.e., more cytochrome c binding) and lower 2 hr. survival in LL-37, there are likely additional residues (e.g., arginine) or regions within the EL of VraG that contribute to the phenotypic differences.

### Biochemical confirmation of the interaction between GraS and EL of VraG

**Fig 5:**
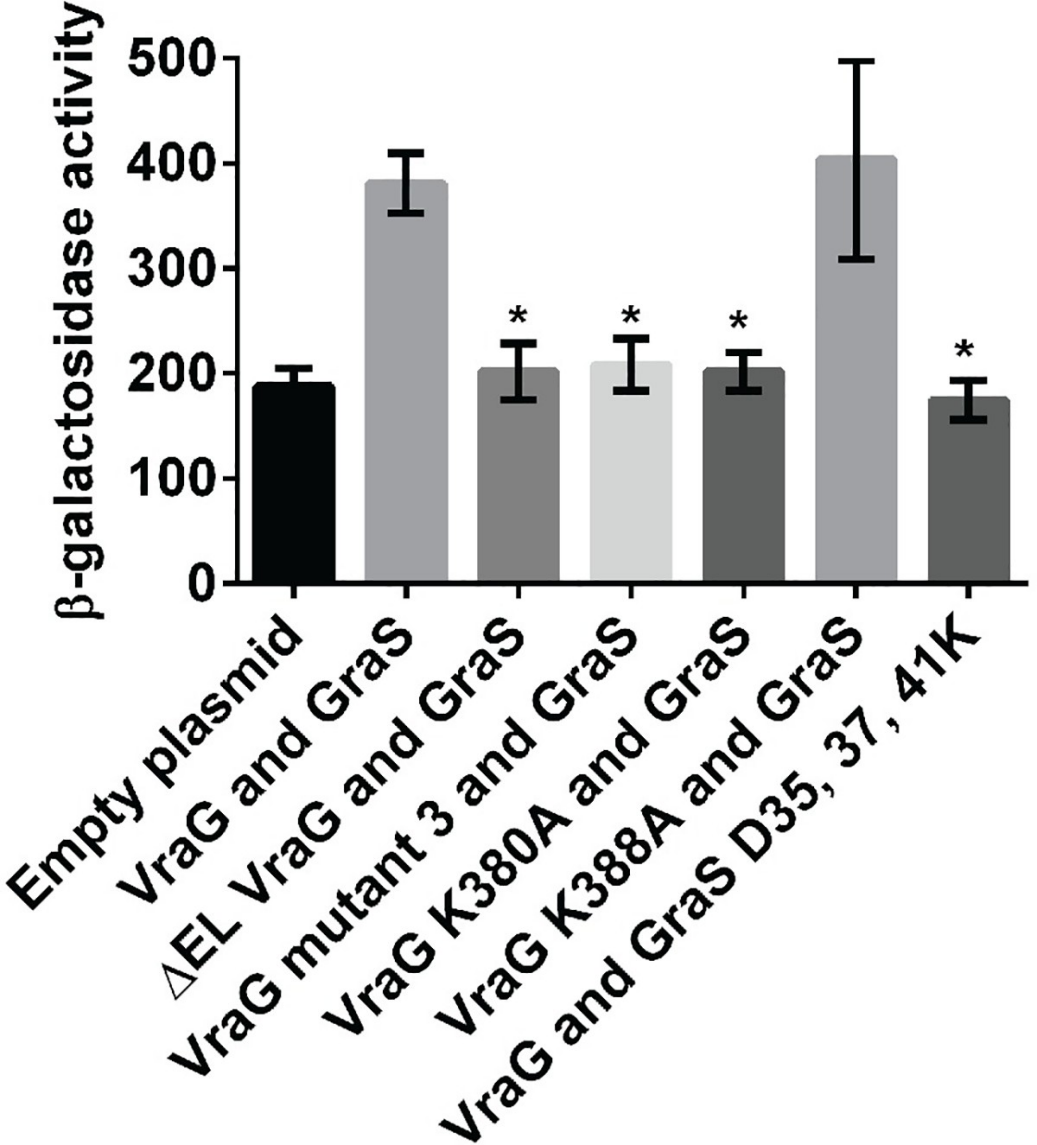
Bacterial two-hybrid analysis of the interaction between GraS and EL of VraG. Interactions between GraS and assorted VraG mutant proteins were assessed by bacterial two-hybrid analysis. The interaction was assessed by β-galactosidase activity with ortho-Nitrophenyl-β-galactoside (ONPG) as the substrate. The empty plasmids were used as the negative control. Various VraG mutant proteins were used to assess interaction with native GraS. In addition, native VraG was deployed to ascertain interaction with GraS with the D35-37-41K mutation, using GraS as a positive control. The data are derived from three biological replicates conducted on the same day. Technical triplicates were used for each assay. The asterisks * represent p <0.05 vs. wild type VraG and GraS, using one-way ANOVA test.

We also verified our genetic analysis with biochemical confirmation of the interaction between GraS and assorted VraG EL mutant proteins, using bacterial two-hybrid analysis in *E*. *coli* strain DHT1 [27]. As shown in Figs 5 and S4, the native VraG interacted with GraS significantly over the basal level as exemplified by the vectors alone. An EL deletion of VraG reduced the interaction to that of the basal level of the empty vectors. Likewise, mutant 3 protein containing K380A and K388A mutations in VraG also failed to associate with GraS. Corresponding to the genetic analysis, VraG with the K380A mutation did not interact with GraS whereas the K388A mutation in VraG did. We also performed the converse experiment where we found that GraS with the D-35-37-41K EL mutation did not associate with VraG whereas the native GraS did.

### Ability of specific EL peptides to alter survival of ΔEL *vraG* mutant in 2 hr. exposure assay to LL-37

**Fig 6:**
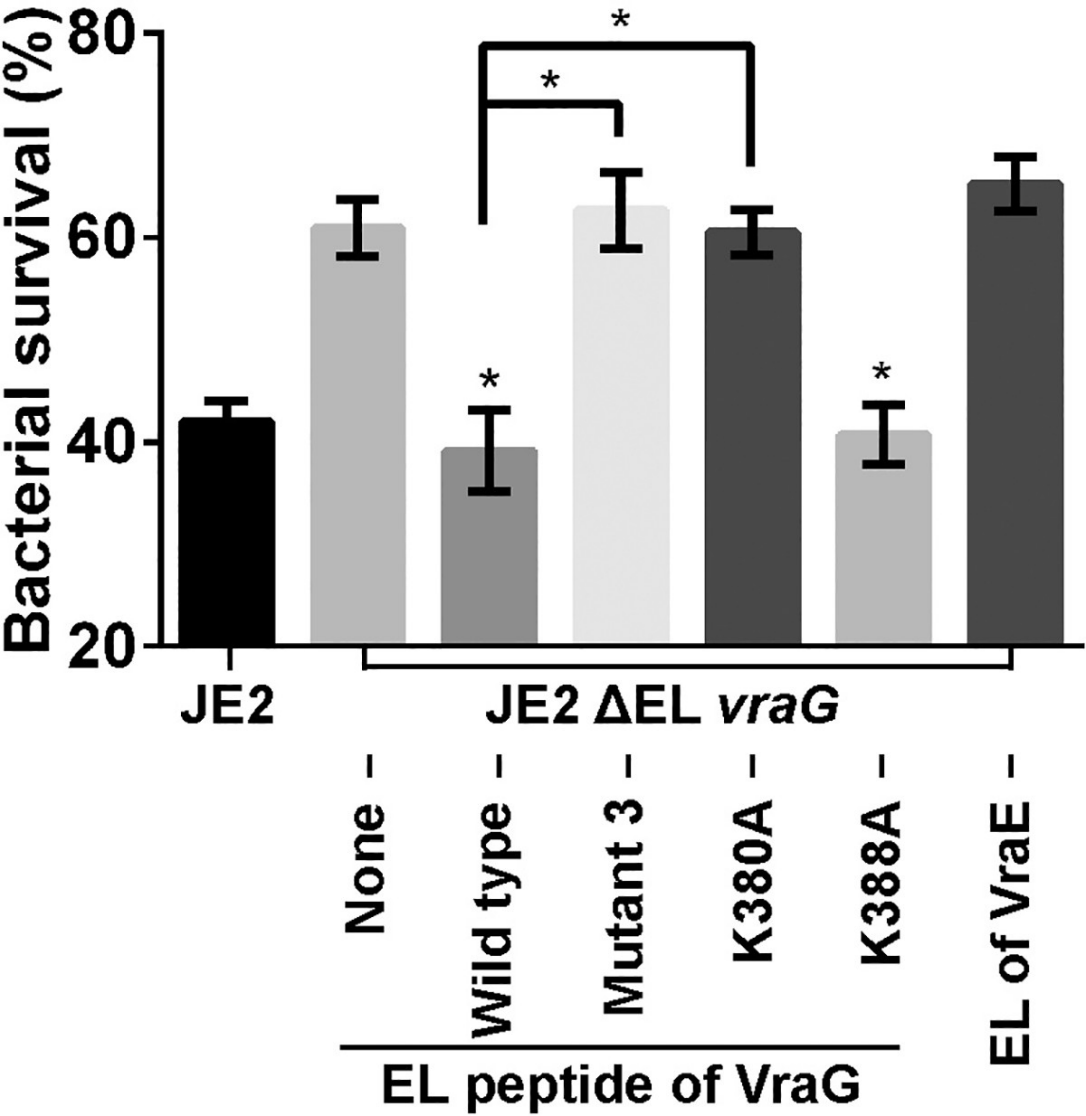
2 hr. killing assay of ΔEL *vraG* mutant in LL-37 with exogenous EL peptides. ΔEL *vraG* mutant cells of JE2 grown to the mid-log phase were incubated with 300 ng of assorted EL peptides of VraG (about 60 nM with 2 X 10^5^ cells), followed by treatment with 2.5 μg/m of LL-37. Lanes counting from left: 1) JE2; 2) ΔEL *vraG* mutant without EL VraG peptide; 3) ΔEL *vraG* mutant with native EL peptide; 4) ΔEL *vraG* mutant with mutant 3 EL peptide (K380A & K388A); 5) ΔEL *vraG* mutant with K380A EL peptide; 6) ΔEL *vraG* mutant with K388A EL peptide; 7) ΔEL *vraG* mutant with EL of VraE. The two lower asterisks * represent p < 0.05 of ΔEL *vraG* mutant with wild type or K388A peptide vs. ΔEL *vraG* mutant with no peptide. The two upper astericks * denote p<0.05 of JE2 ΔEL *vraG* mutant with mutant 3 or K380A EL peptide vs. ΔEL *vraG* mutant with wild type EL peptide. Analysis was done by ANOVA.

We surmised that the EL peptide of VraG, as a distinct domain of the permease, can interfere with GraS-mediated sensing of HDP in a ΔEL *vraG* mutant. We reasoned that exogenous EL peptide, in conjunction with the ΔEL *vraG* mutant which may provide membrane-membrane interaction, would associate with GraS to interfere with GraS-mediated signaling with LL-37, thus leading to reduced survival. For this assay, we exposed the ΔEL *vraG* mutant of JE2 to LL-37 in a 2-hr. killing assay in the presence of wild type or mutated EL peptides. The peptides were expressed in *E*. *coli* as a Histag protein followed by cleavage with TEV protease to yield purified peptides the authenticity of which was verified by MS/MS (S6 Fig). Titration studies revealed that ~250 *ng* of wild type EL peptide was optimal for interference of the survival assay with the ΔEL *vraG* mutant in LL-37 (S7 Fig). Using ~300 *ng* of peptides, we compared the ability of individual peptide to alter survival of the ΔEL *vraG* mutant in LL-37. As shown in Fig 6, the wild type EL peptide reduced survival of the mutant from 60 to ~40%. In contrast, peptides that were predicted not to bind GraS including mutant 3 peptide and K380A peptide enhanced survival, likely due to increase GraS activation whereas the K388A control peptide did not. Collectively, these data are consistent with the bacterial two hybrid assay for the interaction between EL and GraS (Fig 5).

### The specificity of EL of VraG in interaction with specific HDPs

**Fig 7:**
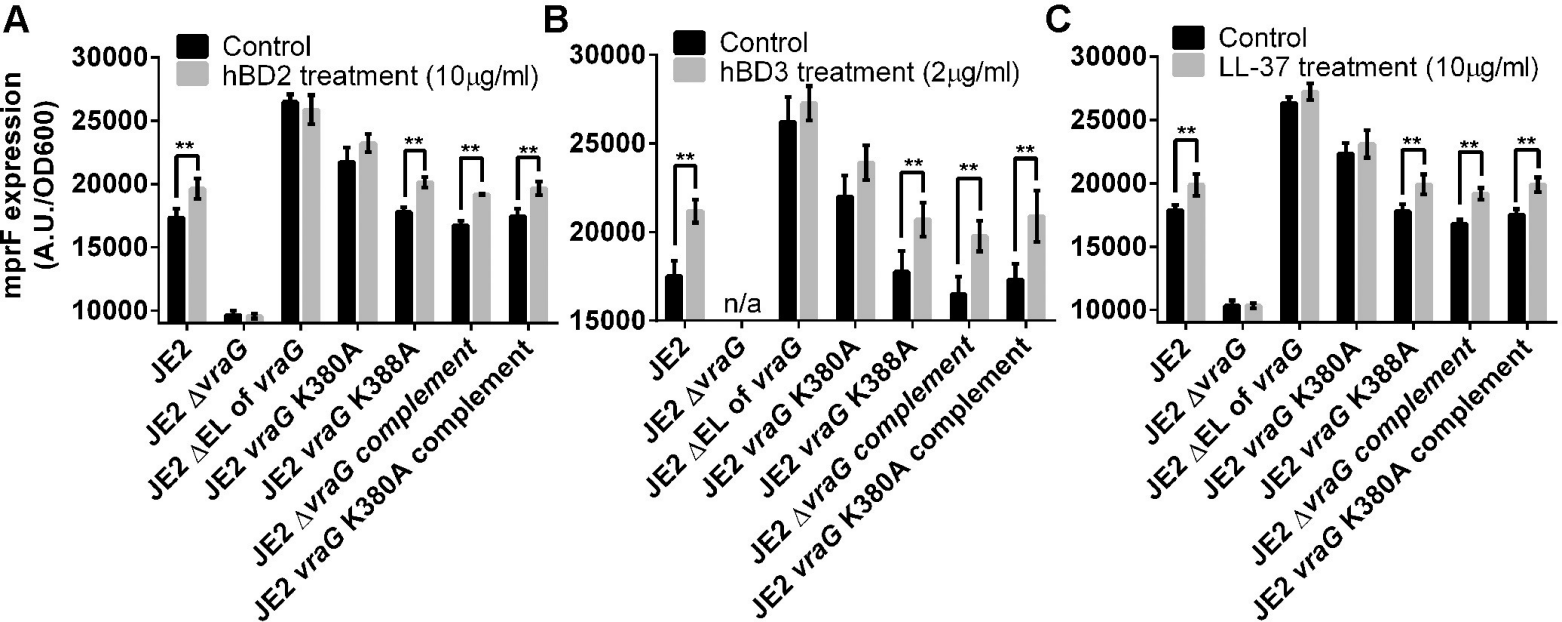
The ΔEL *vraG* mutant is insensitive to HDPs for *mprF* expression. Cells grown to OD_600_ of 0.7–0.8 were divided into two treatment groups each, including treatment of h-BD2 **(A)**, h-BD3 **(B)** or LL-37 **(C)** and water as a control. After 30 min, we monitored OD_600_ (cell growth) and fluorescent intensities of the cells. The results were obtained from three biological replicates. * indicates p < 0.01, using the Student-t test. In the case of Δ*vraG* with h-BD3, cell growth was impaired so that the results were excluded.

The above data supported the notion that the EL of VraG is crucially involved in GraS-mediated signaling, leading to interference in *mprF* expression to alter the surface positive charge. Remarkably, deletion of the EL of VraG appeared to fully activate GraS with and without PMB (S2 Fig). This finding led us to wonder if the EL of VraG dictates interaction with specific HDPs. To satisfy this curiosity, changes in *mprF* expression were measured with prototypic HDPs from PMNs (LL-37) or epithelial tissues (hBD-2 and hBD-3). As shown in Fig 7, all three HDPs enhanced the induction of *mprF* expression in the parent strains and control samples (K388A mutant, K380A complemented strains) whereas the ΔEL *vraG* mutant and, to a lesser degree, the K380A mutant appeared to be much less sensitive to the HDPs treatment. These results indicate that the ΔEL of *vraG* and K380A mutants lifted the restraints in regulating GraS signal transduction, regardless of HDPs.

## Discussion

One avenue for bacterial evasion of HDPs is driven by TCS that enable the bacteria to sense and respond to these cationic but bactericidal molecules. Among the ~2000 histidine kinases from 350 species of Gram-positive bacteria, Proteobacteria and Actinobacteria is a subset called Intramembrane histidine kinase (IM-HK), with each histidine kinase comprising two transmembrane helices framing a very short extracellular loop (<10 residues) for sensing. A large number of IM-HKs including GraS have been found to lie adjacent to ABC transporters genetically linked to the TCS [37]. In the case of *S*. *aureus*, *graRS* have been shown to control the expression of *vraFG* which lies downstream of the TCS and encodes an ATPase-dependent efflux pump without any extracellular nucleotide binding moiety. An important question is how GraS in *S*. *aureus*, as a member of the IM-HK, responds to diverse HDPs (e.g. resistant to hBD-2 but sensitive to h-BD3) with its 9-residue EL which is critical to HDP sensing as supported by extensive mutagenesis studies [14, 36].

Traditionally, ABC type efflux pumps have been known to confer resistance to various antibiotics by discharging the toxic materials out of cells using ATP hydrolysis as the energy source [38, 39]. However, this mode of resistance has not been adequately explained for HDPs because these cationic molecules insert into the bacterial membrane to form pores without ever reaching the cytosol. In prior studies, it has been stated that the membrane permease VraG may play a role as an accessory protein for HDP signaling [11]. While the BceRS-BceAB system for bacitracin resistance in *B*. *subtilis* suggests that the membrane permease BceB is the primary sensor for bacitracin [40, 41], we believe that the GraRS-VraFG system in *S*. *aureus* currently under study differs from this observation since mutagenesis studies in the EL of GraS [14, 36] and that of VraG in this study imply a role for both EL in sensing (Fig 5).

In this study, we showed that the membrane permease VraG contributes to GraS-mediated sensing based on the following observations: 1) bacterial 2-hybrid studies in our lab as well as others [11] showed that GraS interacts with VraG; 2) the *vraG* mutant phenocopied the *graS* mutant with respect to reduced *mprF* activation, more binding to positively charged dye cytochrome c and decreased survival in 2 hr. killing assay with LL-37; 3) deletion of the unique EL in VraG led to higher *mprF* activation than the parent, with and without PMB, accompanied by expected *mprF* activation, enhanced surface positive charge and survival upon 2 hr. exposure to LL-37; 4) bacterial two hybrid studies have divulged the role of EL, in particular the positively charged K380, in interaction with GraS; 5) likewise, D35-37-41 in the EL of GraS appears to be seminal in its association with VraG.

Using the Δ*vraG* mutant as the negative control, we noticed the dichotomy of overnight MIC from rapid sensing of PMB, which is defined by transient *mprF* expression (within 2 hr. of PMB exposure), surface positive charge and 2 hr. killing assay with LL-37, among various EL mutants (ΔEL *vraG* mutant, mutant 3 and K380A mutant) and parent. Instead of having MIC tracking *mprF* expression, we found that the ΔEL *vraG* mutant has an MIC of 16 μg/ml, close to the Δ*vraG* mutant at 8 μg/ml, with the former exhibiting higher *mprF* expression and the latter showing lower *mprF* level that is unresponsive to PMB induction. However, mutant 3 and K380 mutant, which showed increased *mprF* expression vs. parent JE2 (Fig 4A), have MIC at 32 μg/ml, much higher than the Δ*vraG* mutant with low *mprF* expression (S3 Table). Importantly, *mprF* expression data in the ΔEL *vraG* mutant, mutant 3 and K380A mutant paralleled the 2-hr. killing assay with LL-37, correlating higher survival with increased *mprF* expression under induction (S3 Table) and augmented surface positive charge. As signaling is an inductive event with a short response time window (30 min to 2 hr. for *mprF* expression), we propose that *mprF* expression, cytochrome c binding and 2 hr. survival assay with LL-37 correlate with signaling with HDPs whereas MIC is an overnight incubation process that reflects the combination of efflux activity and to a lesser extent, residual signaling activity that may modify the cell surface positive charge. Of concern is whether any of our mutations would damage the integrity of the membrane permease VraG. As compared to the Δ*vraG* mutant where the loss of VraG resulted in low MIC to PMB (at 8 μg/ml) and significantly diminished *mprF* expression without any induction capacity or alteration of growth (S3 Fig), it can be observed that the ΔEL *vraG* mutant exhibited increased sensing to cationic peptides vs. the parent (S3 Table), thus suggesting functional integrity and expression of the VraG protein. We are also careful in our deletion construct as only 180-residue from the EL were removed, leaving 10 residues on each side to preserve better transition to adjoining membrane segments. Finally, we have HA-tagged each version of the VraG variants and showed by Western blot that the native and mutated VraG proteins are expressed (S5 Fig). With regard to the alteration in MIC in the ΔEL *vraG* mutant, it is plausible that prolonged exposure to PMB as found in MIC studies may have led to other damages to result in lower MIC of the ΔEL *vraG* mutant vs. the wild type (16 vs. 128 μg/ml).

*In silico* analysis have revealed that the membrane permease VraG comprises a large 200-residue EL between the 7^th^ and 8^th^ transmembrane segments. However, we did not identify any obvious structural features from this analysis. Recognizing that the 9-residue EL of the GraS, as the sensing component [36] of the TCS responsible for HDP sensing, is probably too short to recognize divergent HDPs on its own, we speculate that the EL of GraS likely interacts with the EL of the membrane permease VraG. Deletion studies of the EL in VraG yielded the surprising result that *mprF* expression, which was normally driven by the presence of PMB, was enhanced in the ΔEL *vraG* mutant vs. parent/complemented mutant; in addition, the ΔEL *vraG* mutant also displayed enhanced surface positive charge, improved 2 hr. survival in LL-37 as well as greater survivability in 1 hr. killing assay with PMNs (Fig 2). These data suggested that the EL of VraG may interfere with the sensing function of the 9-residue EL GraS, resulting in enhanced *mprF* expression in the ΔEL *vraG* mutant. In addition, the ΔEL *vraG* mutant, comprising mainly of membrane segments, is capable of activating GraS as exemplified by enhanced *mprF* expression.

In contrast to the predominance of negatively charged aspartic acid residues in the EL of GraS, we noticed that the EL of VraG harbors proportionately a large number of positively charged lysine residues (16% vs. 10% based on random assignment), with clustering within short stretches of the molecule (Figs 3A and S1). We surmise some of these residues may interact with the negatively charged aspartic acid residues in the EL of GraS which has been shown to be important for HDP sensing [36]. Based on the position and clustering of lysine residues on the EL of VraG, we have constructed 7 mutants that comprise all except one lysine residue positioned within 10 residues adjoining the 7^th^ transmembrane segment. Among these mutants, only mutant 3 (double K380/388A mutant) appeared to have the phenotypic features of the ΔEL *vraG* mutant, showing activation of GraS-signaling, but the level of activation seems to be less than the ΔEL *vraG* mutant as judged by surface positive charge (Fig 4C), 2 hr. survival in LL-37 (Fig 4D) and earlier activation of the *mprF* promoter (Fig 3C). To further pinpoint the lysine residue in mutant 3 responsible for these effects, we analyzed K380A and K388A mutants, showing that K380 in EL of VraG is responsible for modulating sensing of HDP (Fig 4). Interestingly, there remained physiological differences in GraS signaling between ΔEL of *vraG* and K380A mutants as evidenced by: 1) the ΔEL *vraG* mutant was insensitive to PMB for over-induction of *mprF* expression but mutant 3 (double K380/388A mutant) and K380A mutants responded quickly to PMB with elevated *mprF* expression (S2 Fig); 2) a corollary of this insensitivity in induction for the ΔEL *vraG* mutant is that, unlike the parent, the high level of activation of *mprF* via GraS in this mutant is not dependent on the type of HDPs anymore (Fig 7); 3) the ΔEL *vraG* mutant had relatively higher survivability to PMN and LL-37, compared to the K380A mutant (Fig 4D and 4E). These discrepancies might be rooted in the interaction between ELs of VraG and GraS, with deletion of EL in VraG seemingly unmasking the EL of GraS. We surmise that this unmasking event unleashes full activation of GraS, most likely due to absence of masking residues in the EL of VraG. Although our data seem to suggest that the K380A mutation is linked to over-expression of *mprF* in response to PMB (Figs 4 and S2), we postulate that there are additional residues/region or conformation requirement in the EL of VraG that partially restrain the full activation of GraS in the K380A mutant.

**Fig 8:**
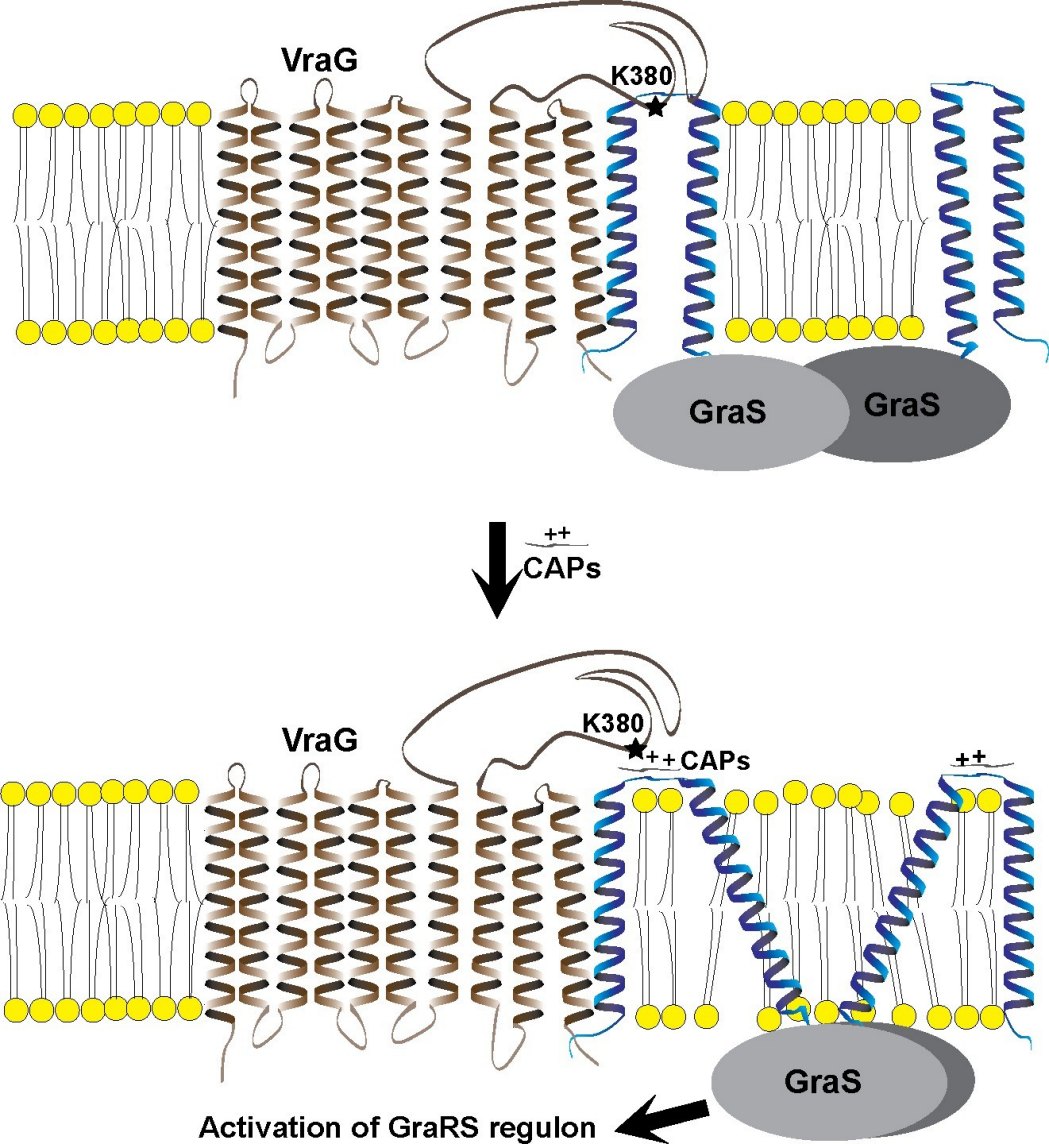
A model of the EL of VraG in modulating HDP-mediated signaling by GraS. The K380 of VraG interacts with the loop of GraS. This tight interaction is loosened by the binding of HDPs to the EL of GraS. The dimerization of GraS would occur under the unlatched condition of GraS in the absence of interference from the EL of VraG, resulting in activation of GraS.

Prior studies in *B*. *subtilis* and other Gram+ organisms have also uncovered the role of transporters in providing resistance to peptide antibiotics [42]. The prototypic example is the BceRS-BceAB system in *B*. *subtilis* where the TCS BceRS controls expression of adjacent *bceAB* encoding a respective ATPase and membrane permease, analogous to the GraRS-VraFG system in *S*. *aureus*. In addition, both BceS and GraS structurally belong to a unique subset (~ 150 genomes) of TCS called Intramembrane histidine kinase (IM-HK), with each sensor kinase comprising two transmembrane helices framing a very short extracellular loop. In contrast to the sensor GraS in *S*. *aureus*, the striking feature of this system is that the sensor kinase BceS lacks any sensor domain and instead relies on BceB for detection of the substrate peptide bacitracin, based on the finding that the *bceAB* promoter lacks any inductive activity in a *bceB* mutant [40, 41]. Importantly, single point mutation in BceB alters bacitracin resistance without affecting its interaction with the sensor kinase, thus indicating separate domains for each of these functions [43]. We speculate that this observation may be prescient for VraG since sensing is enhanced in the ΔEL *vraG* mutant accompanied by a drop in PMB resistance. Like BceS and BceB which forms a sensory membrane complex, we also observed the interaction of GraS and VraG as supported by our bacterial 2-hybrid analysis. However, a distinction can be made here because the BceS-BceB complex is formed in the cell membrane while the EL of VraG is postulated to interact with EL of GraS on the surface of the cell membrane. Nevertheless, the mechanistic basis of signal transduction between the permease BceB and the sensor kinase BceS is not completely defined. In this regard, we propose a model based on our data whereby K380 in the EL of VraG interacts with the negatively charged EL of GraS to hinder its activation. It is conceivable that this charge interaction may restrict the movement of GraS to form a dimer or present a conformation that interferes with GraS activation by phosphorylation (Fig 8). We believe that specific HDP(s) may interfere with this interaction between K380A of VraG and the short sensor loop of GraS that is rich in acidic aspartic residues, thus modulating GraS activation [36]. The transmembrane segments of VraG may also play a role in stabilizing GraS dimerization in the membrane via interaction between neighboring α helices during the signal transduction process since the ΔEL *vraG* mutant, comprising mostly of transmembrane segments, are still capable of enhancing GraS-mediated *mprF* expression. To summarize, GraRS-VraFG in *S*. *aureus* may be a variant of the BceRS-BceAB system in *B*. *subtilis*. However, they do differ in significant ways in that the EL of GraS is an active sensor and that the lysine residue K380 within the EL of VraG interacts with aspartic residues in the EL of GraS to interfere with sensing. Besides charged interaction, there are also membrane-membrane interactions between VraG and GraS since the ΔEL *vraG* mutant, comprising of mainly membrane segments, is capable of sensing cationic peptides with GraS.

In summary, we have unearthed a novel mechanism for HDP signaling capabilities of VraG, a membrane permease which is part of the efflux pump linked to a TCS subset called IM-HK. The EL of VraG which is highly conserved across most MRSA strains, plays a regulatory role by interfering with GraS signaling in the absence of HDPs. We postulate that interference can be disrupted by cognate HDPs to which *S*. *aureus* responds. Enhancing interaction between specific lysine residue(s) or region in the EL of VraG with GraS (e.g., exogenous peptide) would have a strong potential to interfere with the HDP sensing activity of *S*. *aureus*, thus augmenting the ability of the innate immune system (e.g., PMNs) to clear this particular pathogen.

## Material and methods

### Ethics statement

Healthy human neutrophils were obtained with the consent approved by the Committee for the Protection of Human Subjects of the Geisel School of Medicine at Dartmouth (Approval #: STUDY00029984). Formal verbal consent was obtained from the studied subjects. Only adults are included in this study and there were no participation of children in this study.

### Bacterial strains and growth conditions

*Staphylococcus aureus* JE2, a derivative of USA300, was the parental strain (wild type) [24]. To overcome restriction modification (RM) barriers in the *S*. *aureus*, plasmid constructs were passed through *Escherichia coli* IM08B (RM proficient *E*. *coli* for JE2) [26]. *E*. *coli* IM08B strains were grown in Lysogeny Broth (LB) and *S*. *aureus* JE2 strains were cultured in Tryptic Soy Broth (TSB, BD Bacto), Brain Heart Infusion (BHI, BD BBL) or Mueller Hinton Broth (MHB, BD Difco) supplemented with cationic ions; Ca^2+^ 25 mg/L, Mg^2+^ 12.5 mg/L (CAMHB). Autoclave sterilization was used for LB and TSB. Filter sterilization was applied for CAMHB. For plasmid selection, ampicillin (100 μg/ml), chloramphenicol (10 μg/ml) or erythromycin (2.5 μg/ml) were used when necessary. The temperature for bacterial growth was normally set at 37°C, but 42°C (for single crossover) or 30°C (for double crossover) was used for mutant construction.

### Plasmid constructs and bacterial genomic mutations

The genomic information about JE2 strain was obtained from NCBI Genbank database (the accession number CP020619). The sequences of oligonucleotides used in this study were listed in S1 Table. For in-frame deletion and single/multiple nucleotide mutation, the procedures were performed, as described in our previous studies [16, 30]. Briefly, about 1000 bp fragments of upstream and downstream of target sequences were amplified by PCR and linked by 1) SOEing PCR or 2) restriction enzyme cleavage and T4 DNA ligation. These genetic fragments (~2000 bp) were inserted into thermosensitive vector pMAD via restriction: *Bam*HI for single digestion, *Bam*HI and *Xma*I for double digestion). The newly constructed plasmids were introduced into *E*. *coli* recipient strain IM08B. After confirming the nucleotide sequences of the plasmids by DNA sequencing, *S*. *aureus* strain JE2 was transformed with the plasmids by electroporation (Biorad, Gene Pulser) followed by homologous recombination procedures with pMAD. The genomic mutants (Tables 1 and S2) were verified by using both PCR and DNA sequencing.

### Bacterial two hybrid assay (BACTH)

We constructed fusions of GraS or VraG EL fragments into pUT18 or pKT25 for C-terminal or N-terminal insertions followed by transformation of dual recombinant plasmids into DHT1 for expression [11]. Transformants were selected with kanamycin (50 μg/ml) and ampicillin (100 μg/ml) on LB agar. After verification with plasmid digestion and sequencing, three separate colonies from each transformants were selected, grown at 30°C, permeabilized with chloroform and 0.1% SDS, processed with ONPG and absorbance measured at 420nm after a 15-min reaction as described [11].

### MIC assay

We performed MIC assay with PMB (Sigma) as described according to the CLSI standard [25]. Briefly, strains were grown in 5 ml cations-supplemented MHB overnight and serially diluted by 10 folds to make the final concentration of cells to 1 × 10^6^ CFU/ml. 100 μl of these diluted samples were mixed with the equal volume of serial dilutions of PMB in 96-well plates. The mixtures were incubated for 18 hours at 37°C. To arrive at the MICs of PMB for samples, we screened at least three biological replicates.

### 2 hr. LL-37 susceptibility assay

Two hour killing assay with LL-37 (Peptides International) was carried out, as previously described with a minor modification [44, 45]. Briefly, Strains from overnight culture were grown to mid-log phase (OD_600_ ~ 0.8) in 10 ml BHI and washed with a mixture of 10 mM KH_2_PO_4_ and 1% BHI. These cells were serially diluted 10-fold to a concentration of 1 × 10^6^ CFU/ml and then mixed with various concentration of LL-37. These mixtures were incubated at 37°C for 2 hr., diluted for predictable and plausible counting and spread on TSA agar followed by incubation at 37°C overnight. The colonies were counted, and the survival rates were calculated from ratios of initial number of cells and the HDPs-treated cells. The most favorable concentrations used in this assay to distinguish effects of mutations were indicated in the figures. These assays were performed at least three times, and representative data in technical replicates are presented in the figures.

### Cytochrome c binding assay

Strains from overnight culture were grown in 10 ml CAMHB for about 2.5 hrs. until OD_650_ reached 0.5–0.8. These cells in the exponential phase were washed with MOPS, pH 7.0 and resuspended in 500–1000 μl MOPS. The cell concentrations were adjusted to OD_650_ of 3.0 with MOPS using a biophotometer plus with the light beam of 1.0 mm wide and 1.5 mm height (Eppendorf). These samples were spun at 5000 × g for 2 min and resuspended in 300 μl of 0.25 mg/ml cytochrome c solution (sigma) as previously described [34]. These mixtures were incubated at room temperature for 10 min followed by centrifugation at 5000 × g for 2 min to pellet the cells. The supernatants were removed and measured at A_530_ using Tecan plate reader (Infinite M1000 pro). To calculate the cytochrome c binding ratios, we subtracted the estimated values (samples) from the completely unbound values (cytochrome c without cells) and converted into a percentage. The values were obtained from at least three biological replicates.

### Fluorescence assays of GFPuvr driven by the *mprF* promoter

To construct promoter fusion reporter plasmids, we took advantage of plasmid pALC1484 harboring GFPuvr, previously used in our studies [31]. The information of *mprF* and *dltA* promoters were obtained from several studies [46, 47] and the sequences of oligonucleotides for this plasmid construction were listed in S1 Table. Each promoter sequence was amplified by PCR and ligated into pALC1484 double digested with *Eco*RI and *Xba*I. The constructed plasmids were introduced first into IM08B and then into JE2 and its mutants. The sequences of the plasmids were confirmed by PCR and DNA-sequencing. The mutants were grown overnight with chloramphenicol and diluted in CAMHB (1:100 or 1:1000 dilution). OD_600_ and fluorescent intensities (excitation wavelength at 487 nm, emission wavelength at 511 nm) were manually measured every hour for 6 hours by using a spectrophotometer and Tecan M1000 Pro plate reader (fixed gain 120 or 145, z-position of 20,000 μm and flashes of 50 with 400 Hz). The results were calculated from three biological replicates with two technical replicates. To investigate the increments of the GFPuvr expression with PMB or HDPs (Peptides International) induction, we compared the fluorescent intensities of cells at mid-log phase with and without 32 μg/ml PMB treatment or 2 μg/ml hBD3 or 10 μg/ml LL-37 or 10 μg/ml hBD2 at 30 min interval from three independent biological replicates. The relative expression (RE) of *mprF* transcript was decided by dividing A.U. by OD600 to normalize the cell density. The Δ (AU/OD600) was calculated by subtracting RE at 0 min from RE at 30 min.

### PMN assay

Heparinized whole blood was diluted 1:1 in RPMI and centrifuged over Ficoll gradient (GE Healthcare). The erythrocyte pellet was diluted 1:1 in RPMI and the neutrophils were separated by 5% dextran sedimentation at 4°C for 1 hour. Following red blood cell lysis using 1X BD Pharm Lyse (BD Bioscience), neutrophils were resuspended in RPMI with 10% autologous serum [48]. For bacterial preparation, strains grown overnight were diluted 1:500 in CAMHB and freshly re-grown to an OD_600_ of 0.8. After washing with PBS, bacterial cells were opsonized with 5% autologous serum in RPMI with L-glutamine for 30 min at 37°C. The bacteria were diluted to 1 × 10^7^ CFU/ml and 250 μl of bacteria was mixed with equal volume of PMN at ~2.5 × 10^6^ cells/ml and incubated at 37°C for 1 hour. Depending on the amount of purified PMN, MOI slightly varied (ranging from 4 to 8). To lyse the PMN to enable counting, 0.1% Triton X-100 was added to the mixture, followed by serial dilutions. 100 μl of these diluted samples were spread on TSA and incubated overnight. The survived rates were determined from ratios of initial number of non-treated cells and PMN-treated cells. Biologically independent experiments were performed to confirm if the results were consistent.

### Western blot of HA-tagged VraG probed with chicken anti-HA antibody

*S*. *aureus* cells were grown in 100 mL of TSB at 30°C overnight, washed and harvested by centrifuge (4,000 rpm) at 4°C for 15 min. For cell lysis, the bacteria (2 ml) were exposed to 20 μl of lysostaphin (2.5 mg/ml) in 50 mM Tris, pH 7.5, 50 mM NaCl and cOmplete protease inhibitors (Sigma) and benzonase for 30 min on ice. The reaction mixtures were subjected to Bead Beater (30 sec followed by 30 sec of rest for 5 min) to promote complete lysis. The lysate was clarified with a short spin of 2000 g to remove unlysed cells. The supernatant was then spun at 216,000 g at 4°C to obtain the membrane pellet. The pellet was resuspended in 0.1% SDS. After BSA assay, 100 μg of protein (9μl) each was mixed with SDS loading buffer and blotted onto PVDF membrane. The membrane was blocked with 5% skim milk, washed, and probed with 1:1000 dilution of primary chicken anti-HA antibody (Abcam) followed by goat anti-chicken antibody conjugated to alkaline phosphatase (1:5000 dilution, Jackson ImmunoResearch). The membrane was then developed with NBT/BCIP substrate. Densitometric analysis was conducted by using ImageJ (NIH).

### EL peptide expression and purification

Cells (BL21(DE3) with pET14 TEV::EL VraG or C43(DE3) with pET14 TEV::EL VraE) were grown overnight in 5 ml LB with 100μg/ml ampicillin at 37°C followed by 1:500 dilution in 500 ml of LB with antibiotics. Upon reaching the mid-log phase, IPTG was added to a 0.5 mM concentration followed further incubation at 18°C overnight. Cells were harvested and resuspended in 10 ml of lysis buffer (50mM Tris-Cl, pH 7.5, 500mM NaCl, 20mM Imidazole, 5mM MgCl_2_, 2mM β-mercaptoethanol, cOmplete protease inhibitor and 10% glycerol) and lysozyme added (final conc:1mg/ml). After adding benzonase (25 U/ml), the lysate was sonicated on ice at 50% duty cycle for 5 min (20 sec/ 40 sec break for 5 min, output:100%) and then incubated on ice for 30 min. The lysate was spun (>12,000 rpm) and the supernatant containing the peptide was applied to Cobalt resin and incubated at 4°C for 1–2 hrs. The resin was rinsed with washed buffer (50mM Tris-Cl, pH 7.4, 500mM NaCl, 40mM Imidazole, 2mM BME, 5mM MgCl_2_ and 10% glycerol) and eluted with elution buffer (50mM Tris-Cl, pH 7.4, 100mM NaCl, 500mM Imidazole, 1mM BME, 10% glycerol). After dialysis exchange (50mM Tris pH 7.4, 50mM NaCl, 0.5mM EDTA, 1mM BME, 10% glycerol), the sample was concentrated in spin column to 200 μl. About 15 μg of protein was mixed with 1 μl of 10,000U/ml TEV at 4°C overnight. The following day, the sample was mixed with 1 μl of Ni-NTA resin equilibrated with 50mM Tris, 50mM NaCl, 1mM Imidazole. After 30 min, the mixture was centrifuged to obtain the peptide without the Histag in the supernatant. Protein concentration was determined with a Bradford assay. All peptides were verified by SDS-PAGE and then authenticated by MS/MS at the Vermont biomedical Research Network (VBRN) Proteomic Facility.

### 2 hr. killing assay of ΔEL *vraG* mutant in LL-37 with exogenous EL peptides

The ΔEL *vraG* mutant and the parental strain JE2 were grown in BHI at 37°C to mid-log phase. The cultured was then diluted in 10 mM phosphate buffer pH 7.4 in 1% BHI to yield ~10^7^ CFU/ml. Samples for EL variants or control samples (no peptide or wild type peptides) are prepared as follows: EL VraG peptide variants in 50mM Tris pH 7.4 were mixed with 150 μl of 10mM phosphate buffer with 1% BHI and 20 μl of ΔEL *vraG* mutant (10^7^ CFU/ml). JE2 or ΔEL *vraG* mutant without any peptides was used as controls. The samples were then incubated at room temperature for 15 min to give enough time for EL to bind to bacterial cells. LL-37 was then added to a final concentration of 2.5 μg/ml followed by incubation at 37°C for 2 hrs. Samples were then diluted and enumerated.

## Supporting information

S1 Fig Illustration of the VraG membrane structure. TMHMM and TOPCONS servers for prediction of membrane structure of VraG were used. The loops and membrane helices were displayed as narrow lines and rectangular shapes, respectively. In. stands for intracellular or cytoplasmic loop. Ex. indicates extracellular loop. Lysine residues in the long extracellular loop are marked with asterisks. The dashed line illustrates the EL deletion.
(TIF)

S2 Fig Expression of *mprF* promoter linked to GFPuvr reporter upon PMB treatment. The OD600s and A.U. with a fixed gain value at 145 in various strains were measured at two time points (before and 30 min after the addition of 32 μg/ml PMB). The results were obtained from three biological replicates. ** indicates p < 0.05 with the Student-t test.
(TIF)

S3 Fig Growth curves of assorted *vraG* strains with mutations of lysine to alanine in the extracellular loop. The growth curves were monitored by measuring OD600 of each mutant every hour for 6 hours. The error bars (S.D.) were calculated by three biological replicates.
(TIF)

S4 Fig Bacterial two hybrid assay on plates. Colonies of DHT1 transformed with various plasmids grown from LB plates were streaked on LB plates with 0.5mM IPTG and 40 μg/ml X-gal, followed by 5 days incubation at room temperature. **(A)** (+) zip: DHT1 with pKT25-zip and pUTC-zip (leucine zipper) | (-): DHT1 with pKT25 and pUT18 | WT vraG WT graS: DHT1 with pKT25::*vraG* and pUT18::*graS* | ΔEL VraG WT GraS: DHT1 with pKT25:: ΔEL *vraG* and pUT18::*graS* | VraG mutant 3 WT GraS: DHT1 with pKT25::*vraG* mutant 3 and pUT18::*graS* | VraG K380A WT GraS: DHT1 with pKT25::*vraG* K380A and pUT18::*graS* | VraG K388A WT GraS: DHT1 with pKT25::*vraG* K388A and pUT18::*graS*. **(B)** (+) zip: DHT1 with pKT25-zip and pUTC-zip (leucine zipper) | (-): DHT1 with pKT25 and pUT18 | WT vraG WT graS: DHT1 with pKT25::*vraG* and pUT18::*graS* | WT VraG GraS D35,37,41K: DHT1 with pKT25::*vraG* and pUT18::*graS* D35, 37, 41K | WT VraG GraS D35K: DHT1 with pKT25::*vraG* and pUT18::*graS* D35K | WT VraG GraS D37K: DHT1 with pKT25::*vraG* and pUT18::*graS* D37K | WT VraG GraS D41K: DHT1 with pKT25::*vraG* and pUT18::*graS* D41K.
(TIF)

S5 Fig Western blot analysis for HA-tagged VraG. 8% SDS-PAGE stained with AcquaStain (above) and western blot detected by NBT/BCIP for primary chicken HA-antibody and secondary goat anti-chicken conjugated to alkaline phosphatase (below). Starting on the left, Bottom Lanes: 1) JE2 wild type;2) JE2 with HA-tag on C-terminal of *vraG;* 3) ΔEL *vraG* mutant with HA-tag; 4) *vraG* mutant 3 (K380A & K388A mutations) with HA-tag; 5) *vraG* K380A mutant with HA-tag; 6) *vraG* K388A mutant with HA-tag. Densitometry was performed on bands representing HA-tag VraG variants: i) wild type JE2, 30532 densitometric units; ii) ΔEL *vraG* mutant, 27538 units; iii) *vraG* mutant 3 (K380A & K388A mutations), 40507 units; iv) *vraG* K380A mutant, 28355 units; v) *vraG* K388A mutant, 32044 units.
(TIF)

S6 Fig Expression of EL VraG variants and VraE peptides. (A) A representative 10% SDS gel stained with AcquaStain for peptide purification (EL VraG). (B) Assorted EL peptides with / without His-tag.
(TIF)

S7 Fig 2hr-killing assay with LL-37 with EL peptide titration. Cells grown to the mid-log phase (OD_600_ ~0.8) were incubated with various concentrations of wild type EL peptides and treated with LL-37. A representative graph with three technical replicates is illustrated. All the samples were treated with 2.5 μg/ml LL-37 except one sample to check if the peptide itself affects the bacterial survivability.
(TIF)

S1 Table A list of oligonucleotides. (DOCX)

S2 Table A list of strains used in the experiments. (DOCX)

S3 Table A summary of mutation effects on MIC, *mprF* activation, cytochrome c binding and 2 hr. survival assay with LL-37. This table shows the summary of MICs of PMB, *mprF* expression, cytochrome c binding and 2 hr. LL-37 susceptibility assays. Expression of *mprF* is relative comparison. For cytochrome c binding, the parent JE2 is set at 100%. For LL-37 assay, survival without LL-37 is set at 100%. The symbols indicate the following: ↑↑ / ↑ / ↓ / ↓↓ (high / moderate / low / considerably low expression.
(DOCX)
