# Supplementary figures and images for: The extracellular loop of the membrane permease VraG interacts with GraS to sense cationic antimicrobial peptides in Staphylococcus aureus
Source: PLoS Pathog. 2021 Mar 1;17(3):e1009338. doi: 10.1371/journal.ppat.1009338 (PMC7951975; doi:10.1371/journal.ppat.1009338)

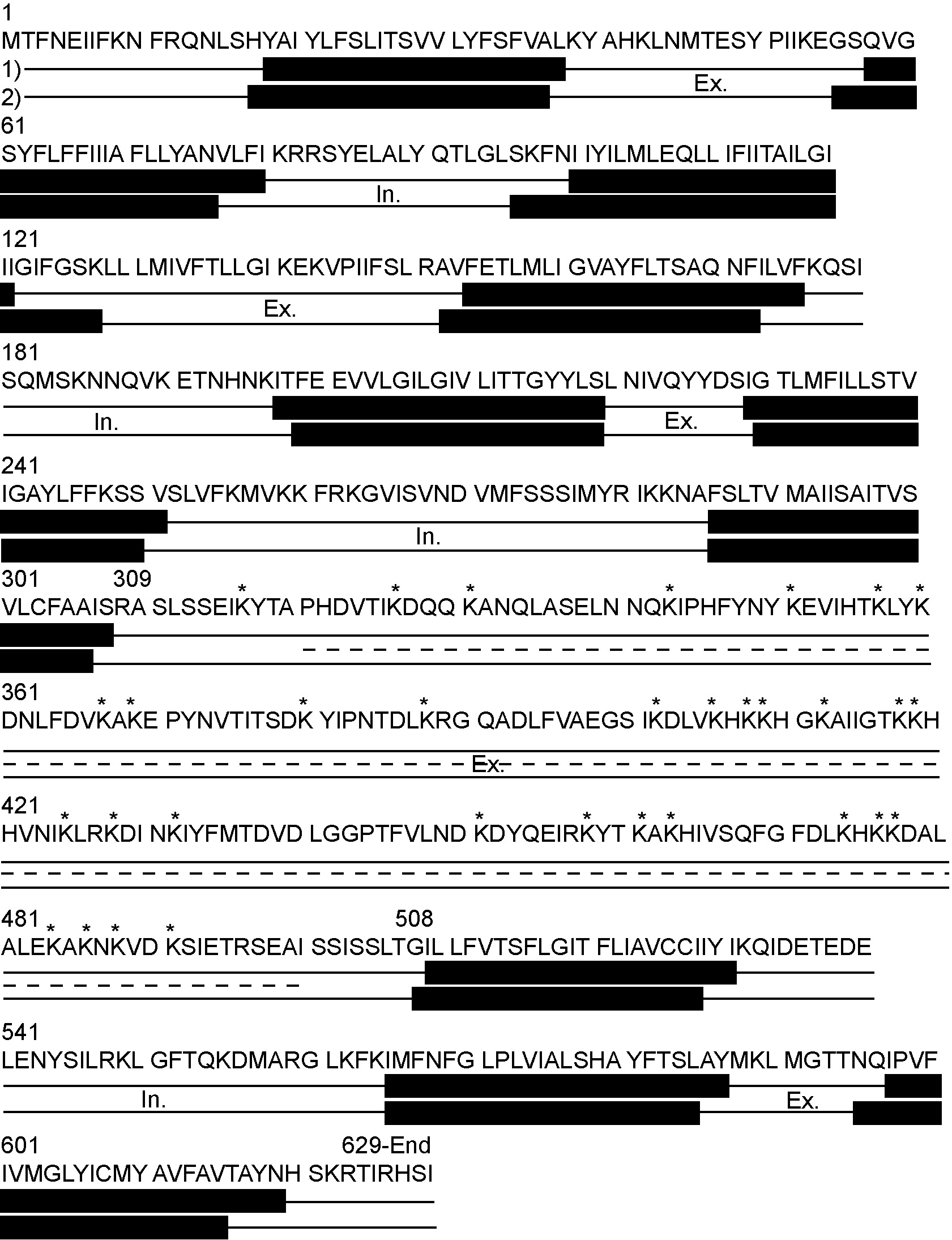

Supplement: S1 Fig — TMHMM and TOPCONS servers for prediction of membrane structure of VraG were used. The loops and membrane helices were displayed as narrow lines and rectangular shapes, respectively. In. stands for intracellular or cytoplasmic loop. Ex. indicates extracellular loop. Lysine residues in the long extracellular loop are marked with asterisks. The dashed line illustrates the EL deletion. (TIF) [file ppat.1009338.s001.tif]

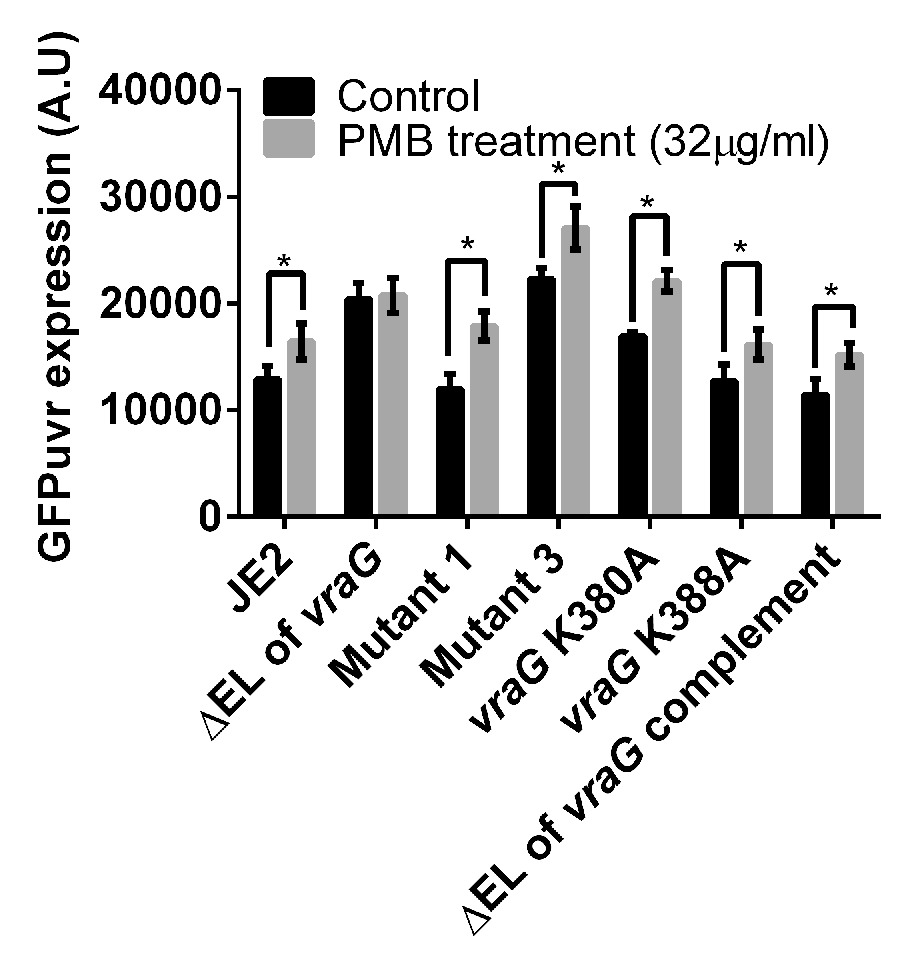

Supplement: S2 Fig — The OD600s and A.U. with a fixed gain value at 145 in various strains were measured at two time points (before and 30 min after the addition of 32 μg/ml PMB). The results were obtained from three biological replicates. ** indicates p < 0.05 with the Student-t test. (TIF) [file ppat.1009338.s002.tif]

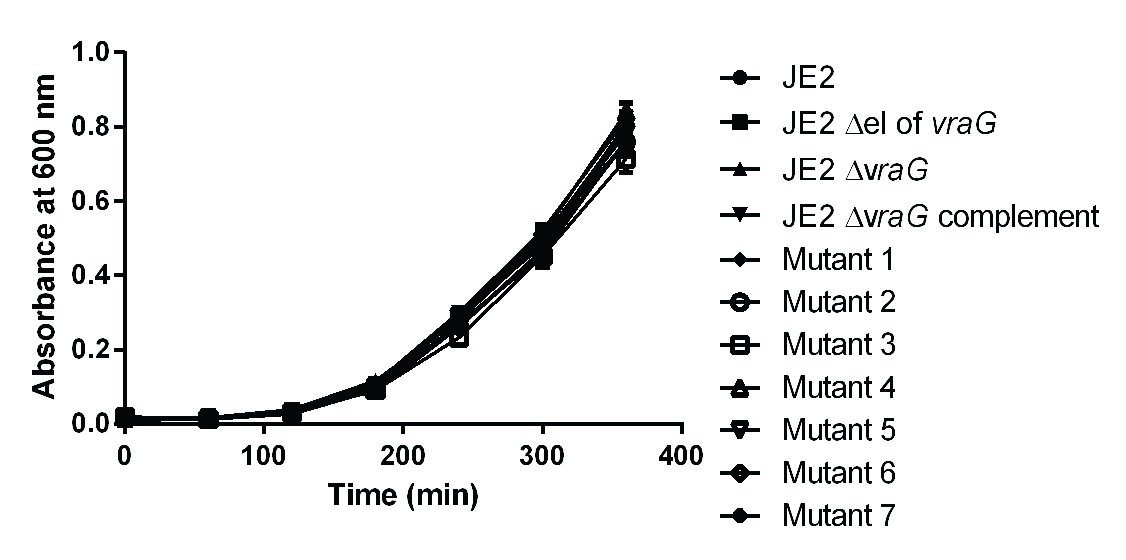

Supplement: S3 Fig — The growth curves were monitored by measuring OD600 of each mutant every hour for 6 hours. The error bars (S.D.) were calculated by three biological replicates. (TIF) [file ppat.1009338.s003.tif]

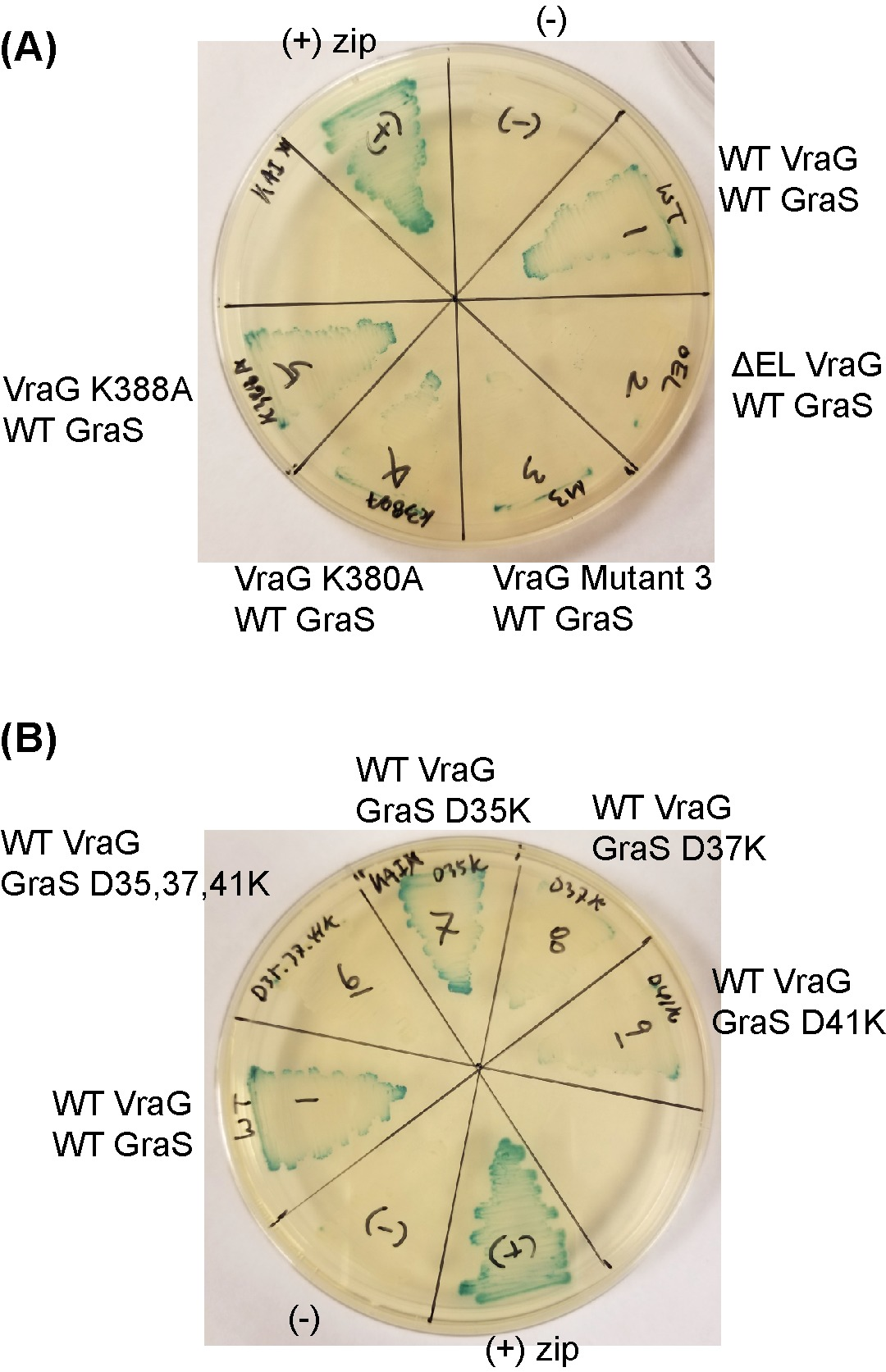

Supplement: S4 Fig — Colonies of DHT1 transformed with various plasmids grown from LB plates were streaked on LB plates with 0.5mM IPTG and 40 μg/ml X-gal, followed by 5 days incubation at room temperature. (A) (+) zip: DHT1 with pKT25-zip and pUTC-zip (leucine zipper) | (-): DHT1 with pKT25 and pUT18 | WT vraG WT graS: DHT1 with pKT25::vraG and pUT18::graS | ΔEL VraG WT GraS: DHT1 with pKT25:: ΔEL vraG and pUT18::graS | VraG mutant 3 WT GraS: DHT1 with pKT25::vraG mutant 3 and pUT18::graS | VraG K380A WT GraS: DHT1 with pKT25::vraG K380A and pUT18::graS | VraG K388A WT GraS: DHT1 with pKT25::vraG K388A and pUT18::graS. (B) (+) zip: DHT1 with pKT25-zip and pUTC-zip (leucine zipper) | (-): DHT1 with pKT25 and pUT18 | WT vraG WT graS: DHT1 with pKT25::vraG and pUT18::graS | WT VraG GraS D35,37,41K: DHT1 with pKT25::vraG and pUT18::graS D35, 37, 41K | WT VraG GraS D35K: DHT1 with pKT25::vraG and pUT18::graS D35K | WT VraG GraS D37K: DHT1 with pKT25::vraG and pUT18::graS D37K | WT VraG GraS D41K: DHT1 with pKT25::vraG and pUT18::graS D41K. (TIF) [file ppat.1009338.s004.tif]

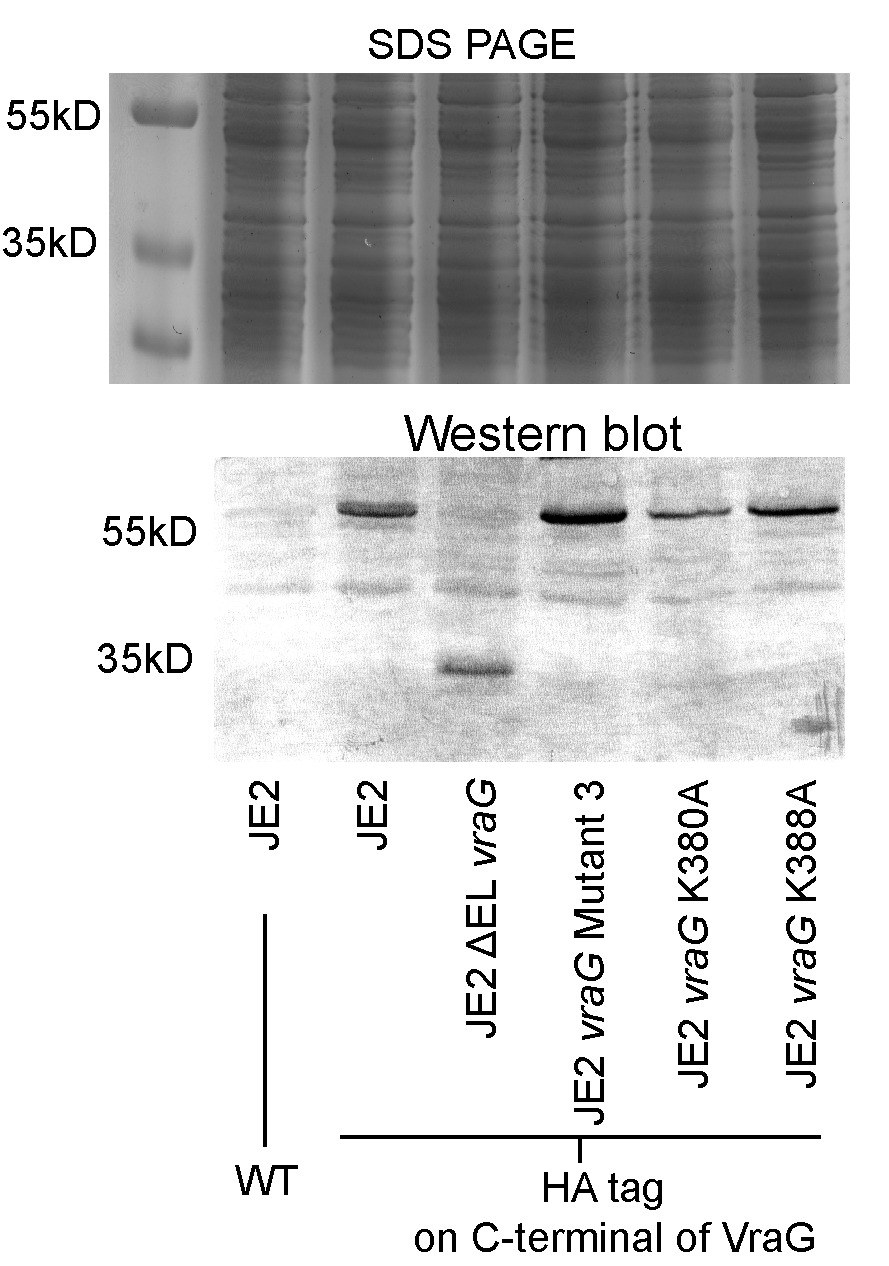

Supplement: S5 Fig — 8% SDS-PAGE stained with AcquaStain (above) and western blot detected by NBT/BCIP for primary chicken HA-antibody and secondary goat anti-chicken conjugated to alkaline phosphatase (below). Starting on the left, Bottom Lanes: 1) JE2 wild type;2) JE2 with HA-tag on C-terminal of vraG; 3) ΔEL vraG mutant with HA-tag; 4) vraG mutant 3 (K380A & K388A mutations) with HA-tag; 5) vraG K380A mutant with HA-tag; 6) vraG K388A mutant with HA-tag. Densitometry was performed on bands representing HA-tag VraG variants: i) wild type JE2, 30532 densitometric units; ii) ΔEL vraG mutant, 27538 units; iii) vraG mutant 3 (K380A & K388A mutations), 40507 units; iv) vraG K380A mutant, 28355 units; v) vraG K388A mutant, 32044 units. (TIF) [file ppat.1009338.s005.tif]

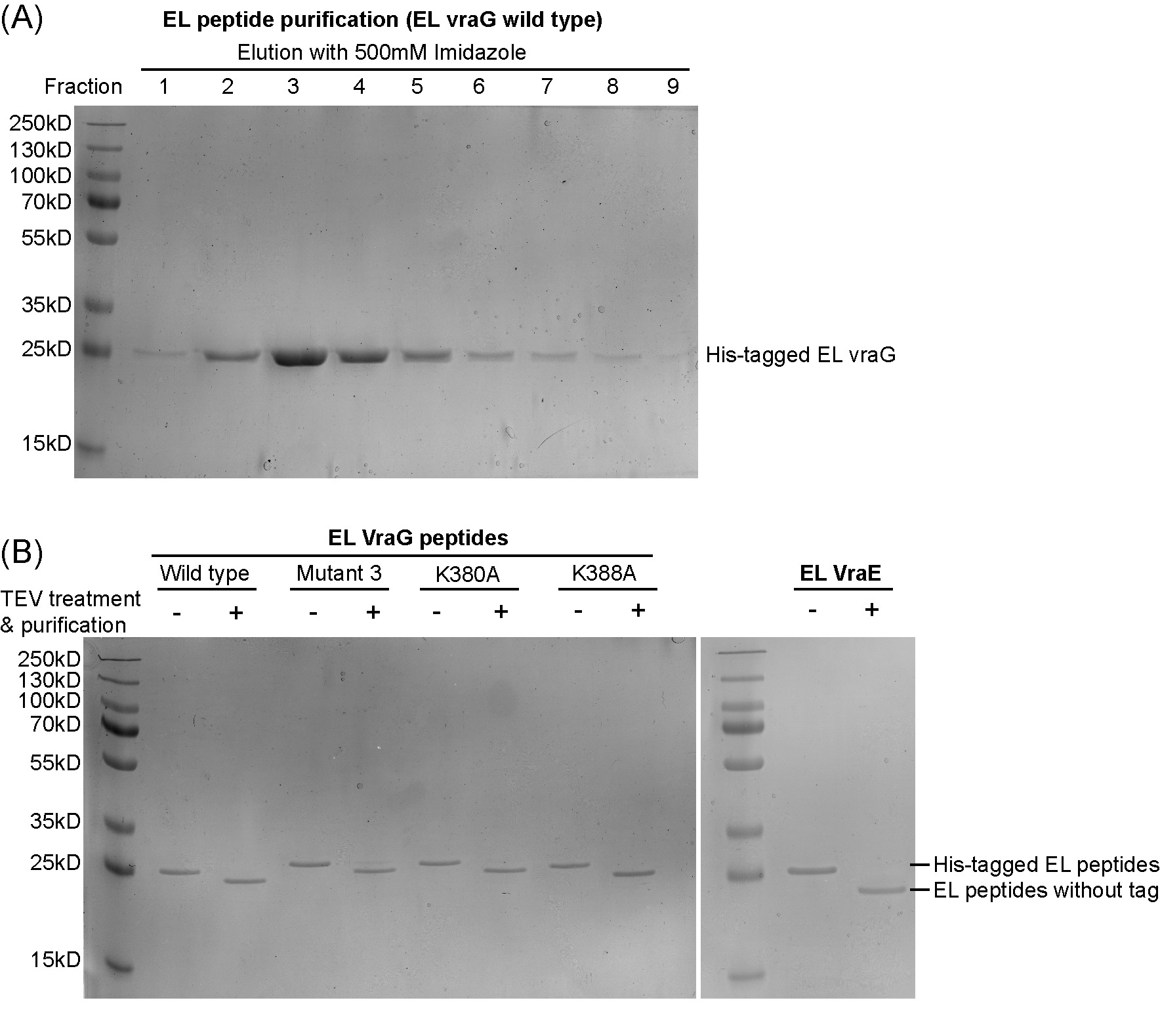

Supplement: S6 Fig — (A) A representative 10% SDS gel stained with AcquaStain for peptide purification (EL VraG). (B) Assorted EL peptides with / without His-tag. (TIF) [file ppat.1009338.s006.tif]

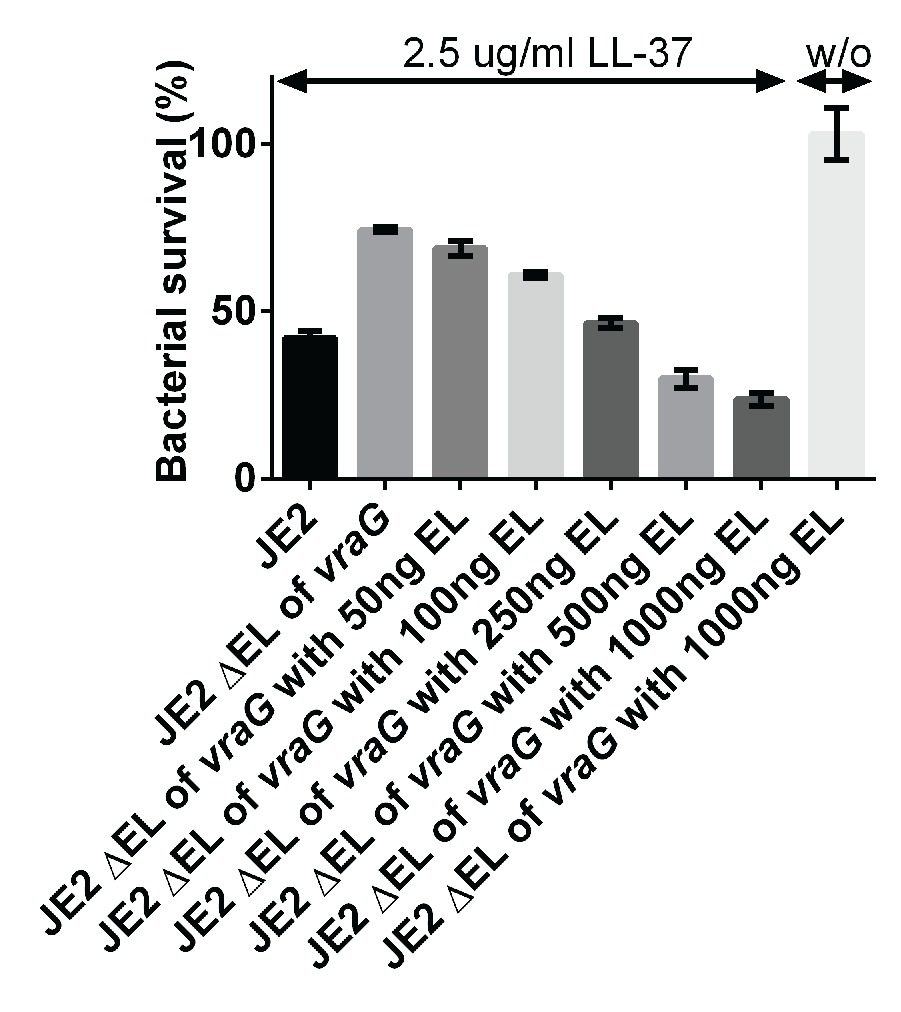

Supplement: S7 Fig — Cells grown to the mid-log phase (OD600 ~0.8) were incubated with various concentrations of wild type EL peptides and treated with LL-37. A representative graph with three technical replicates is illustrated. All the samples were treated with 2.5 μg/ml LL-37 except one sample to check if the peptide itself affects the bacterial survivability. (TIF) [file ppat.1009338.s007.tif]
