# Supplementary material for: The extracellular loop of the membrane permease VraG interacts with GraS to sense cationic antimicrobial peptides in Staphylococcus aureus
Source: PLoS Pathog. 2021 Mar 1;17(3):e1009338. doi: 10.1371/journal.ppat.1009338 (PMC7951975; doi:10.1371/journal.ppat.1009338)
Supplement: S1 Table — (DOCX) [file ppat.1009338.s008.docx]

| Plasmids | Sequence (5’ to 3’, bold: restriction sites) | Ref. |
| --- | --- | --- |
| *graS*_pMAD_Up_F | AAAGGATCCGGTGCGACCGCTCGCAG | [14] |
| *graS*_pMAD_Up_R | AAACCCGGGCCAAAATATCCAGTTCATGCG |  |
| *graS*_pMAD_Down_F | AAACCCGGGGAACGCATGTCGGAAGTGAC |  |
| *graS*_pMAD_Down_R | AAAGGATCCCAATACTACACTCGTAATTAACG |  |
| *vraG*_pMAD_Up_F | AAAGGATCCGGGGACAACTGTCAGATTG |  |
| *vraG*_pMAD_Up_R | AAACCCGGGGGCATAATGTGATAAATTTTGACG |  |
| *vraG*_pMAD_Down_F | AAACCCGGGGCAGTGACGGCTTATAATCAT |  |
| *vraG*_pMAD_Down_R | AAAGGATCCCATCATCAATTGCATCACATAATG |  |
| El of *vraG* pMAD Up F | AAAAAGTCGACCAAAGAAATAATACGTGTACAAAGTGT | This study |
| El of *vraG* pMAD Up R | AAAAACCCGGGTGCAGTATATTTTATTTCACTTGATAAGG | This study |
| El of *vraG* pMAD Down F | AAAAACCCGGGAGCTCAATATCAAGTTTAACCGG | This study |
| El of *vraG* pMAD Down R | AAAAAGGATCCATTTCAAAAATGCCTCACAGTAAA | This study |
| *graS* complement pMAD F | AAAAAACCCGGGAGTGGAAGTTTAGTGAAAAAATATACAGTTA | This study |
| *graS* complement pMAD R | AAAAAAGGATCCATGGCATAATGTGATAAATTTTGACGG | This study |
| *vraG* complement pMAD F | AAAAAACCCGGGTGAAACGACGTCTTCAGGTATG | This study |
| *vraG* complement pMAD R | AAAAAAGGATCCTCTAAAACATCATCAATTGCATCACATAAT | This study |
| *graS*_IDF_F | CGATGAAGGTAGTTTTACCAAAGG | This study |
| *graS*_IDF_R | GACTTGTGAGCCTTCCTTTA | This study |
| *vraG*_IDF_F | GAACGAGGATTTACGTCAACG | This study |
| *vraG*_IDF_R | ACTAATAAGCCGACAGCAAGT | This study |
| IDF_1 for DNA seq | GTTCATCACTTTCAGCAACGA | This study |
| IDF_2 for DNA seq | TAGATCAACGATGAAGGTAGTTTT | This study |
| IDF_3 for DNA seq | AAATTACAAGCGATTTATCGTCGT | This study |
| IDF_4 for DNA seq | TCAAGAAAGAAAACAAGCATTACTA | This study |
| IDF_5 for DNA seq | AATATATGGAACTAAAAAAATGGCACA | This study |
| IDF_6 for DNA seq | TACGAGTGTAGTATTGTATTTTAGCTTTG | This study |
| IDF_7 for DNA seq | GTAAATGATGTCATGTTCTCATCA | This study |
| IDF_8 for DNA seq | CTGTATGTTGCATTATATACATAAAGC | This study |
| pMAD_IDF_F | GTTACGTTACACATTAACTAGACAG | This study |
| pMAD_IDF_R | GAAGAATCATAATGGGGAAGG | This study |
| vraG lysine mutant pMAD F | AAAAAACCCGGGGAGATAATATTTAAAAATTTCCGTCAAAATTTATCAC | This study |
| vraG lysine mutant pMAD R | AAAAAAGGATCCCTGATATTAATTTCAAAAATGCCTCAC | This study |
| *vraG* mutant1 1 | TTAGCTGCTTGTTGGTCTGCAATTGTAACGTCGTGTGGTG | This study |
| *vraG* mutant1 2 | TGCTTGATTGTTTAATTCACTTGCTAATTGATTAGCTGCTTGTTGGTCTGC | This study |
| *vraG* mutant1 3 | GCAGACCAACAAGCAGCTAATCAATTAGCAAGTGAATTAAACAATCAAGCA | This study |
| *vraG* mutant1 4 | GTGAATTAAACAATCAAGCAATTCCTCATTTTTATAATTATAAAGAAGTAATTCATAC | This study |
| *vraG* mutant2 1 | GCCGTATGAATTACTTCTGCATAATTATAAAAATGAGGAATTTTTTGATTGTTTAATTC | This study |
| *vraG* mutant2 2 | TGCCGCTGCTACATCAAATAAATTATCTGCATACAATGCCGTATGAATTACTTCTGC | This study |
| *vraG* mutant2 3 | GCAGAAGTAATTCATACGGCATTGTATGCAGATAATTTATTTGATGTAGCAGCGGCA | This study |
| *vraG* mutant2 4 | TATTTGATGTAGCAGCGGCAGAACCATACAATGTAACAATTACTAGTG | This study |
| *vraG* mutant3 1 | GTGCCAAATCAGTATTAGGGATGTATGCATCACTAGTAATTGTTACATTGTATGGTTC | This study |
| *vraG* mutant3 2 | GATGCATACATCCCTAATACTGATTTGGCACGTGGGCAAGCTGATTTATTTG | This study |
| *vraG* mutant4 1 | CTGCATGTGCCACTAAATCTGCGATAGAACCTTCCGCTACAAATAAATC | This study |
| *vraG* mutant4 2 | TGCTGCCGTTCCTATAATTGCTGCACCATGTGCTGCATGTGCCACTAAATCTGC | This study |
| *vraG* mutant4 3 | GCAGATTTAGTGGCACATGCAGCACATGGTGCAGCAATTATAGGAACGGCAGCA | This study |
| *vraG* mutant4 4 | GGAACGGCAGCACATCATGTTAATATTAAGTTACGTAAAGATATTAATAAAATCTATT | This study |
| *vraG* mutant5 1 | TGCATTAATATCTGCACGTAATGCAATATTAACATGATGTTTTTTCGTTCCTAT | This study |
| *vraG* mutant5 2 | GCATTACGTGCAGATATTAATGCAATCTATTTTATGACAGATGTTGATTTAGGT | This study |
| *vraG* mutant6 1 | TGCTCTTATTTCTTGATAGTCTGCGTCATTTAAGACAAACGTTGGTCC | This study |
| *vraG* mutant6 2 | TGCTGCTGCTGTATATGCTCTTATTTCTTGATAGTCTGC | This study |
| *vraG* mutant6 3 | GCAGACTATCAAGAAATAAGAGCATATACAGCAGCAGCA | This study |
| *vraG* mutant6 4 | ATAAGAGCATATACAGCAGCAGCACATATCGTCTCTCAATTTGGATTC | This study |
| *vraG* mutant7 1 | AAAGCATCTGCTGCATGTGCCAAATCGAATCCAAATTGAGAGACG | This study |
| *vraG* mutant7 2 | TGCATCAACTGCATTTGCCGCTGCTTCTAATGCTAAAGCATCTGCTGCATGTGC | This study |
| *vraG* mutant7 3 | GCACATGCAGCAGATGCTTTAGCATTAGAAGCAGCGGCAAATGCAGTTGATGCA | This study |
| *vraG* mutant7 4 | GCGGCAAATGCAGTTGATGCATCTATTGAAACAAGAAGTGAAGCGATAA | This study |
| *vraG* K380A F | CAATTACTAGTGATGCATACATCCCTAATAC | This study |
| *vraG* K380A R | GTATTAGGGATGTATGCATCACTAGTAATTG | This study |
| *vraG* K388A F | CTAATACTGATTTGGCACGTGGGCAAG | This study |
| *vraG* K388A R | CTTGCCCACGTGCCAAATCAGTATTAG | This study |
| *mprF* promoter pALC1484 F | AAAAAAGAATTCCAATCGTTTCTATGGTAATGAT | This study |
| *mprF* promoter pALC1484 R | AAAAAATCTAGATAATTATTTCTGTTATAAATCAAAATATATCA | This study |
| *dltA* promoter pALC1484 F | AAAAAAGAATTCATTAATGGGACGTGATAATGTT | This study |
| *dltA* promoter pALC1484 R | AAAAAATCTAGAAACTCATTATAAATGAAGTTATTGTGT | This study |
| pALC1484 IDF F | GGCGATTAAGTTGGGTAACG | This study |
| pALC1484 IDF R | CTGACAGAAAATTTGTGCCC | This study |
| VraG pMAD Up_F | AAAGGATCCGAGTCATATCCAATTATAAAGGAAGG | This study |
| VraG HA Tag Up R | AGCGTAATCTGGAACATCGTATGGGTATATGGAATGTCTAATTGTTCGCTTG | This study |
| VraG HA Tag Down F | TACCCATACGATGTTCCAGATTACGCTTAAAATATACAGATGGCTTTCAGTAGAG | This study |
| VraG pMAD Down R for EL | AAAGGATCCCATCATCAATTGCATCACATAATG | This study |
| HA IDF F | CAGCGATTACTGTTTCAGTTC | This study |
| HA IDF R | GCGTAATCTGGAACATCG | This study |
| pKT25 vraG F | AAAGGATCCCACCTTTAACGAGATAATATTTAAAAATTTCC | This study |
| pKT25 vraG R | AAAGAATTCTTATATGGAATGTCTAATTGTTCGC | This study |
| pKT25 IDF F | GATATCGACATGTTCGCCA | This study |
| pKT25 IDF R | GGCGATTAAGTTGGGTAAC | This study |
| pUT18 graS F | AAAGGATCCCAATAATTTGAAATGGGTAGCTTATTTTTTG | This study |
| pUT18 graS R | AAAGAATTCGAAAATGACAAATTTGTCACTTCCG | This study |
| pUT18 IDF F | GTTTCCCGACTGGAAAGC | This study |
| pUT18 IDF R | GACATTAACCTATAAAAATAGGCGTATC | This study |
| pET14b TEV Gibson assembly F | GAAAACCTGTATTTTCAGGGACATATGCTCGAGGATCCG | This study |
| pET14b TEV Gibson assembly R | TCCCTGAAAATACAGGTTTTCGTGATGATGATGATGATGGC | This study |
| pET14b TEV EL vraG F | AAACATATGAGAGCGTCCTTATCAAGTG | This study |
| pET14b TEV EL vraG R | AAAGGATCCTTATCCGGTTAAACTTGATATTGAG | This study |
| pET14b TEV EL vraE F | AAACATATGAAATCAAATACAGATCAAACCCTTAC | This study |
| pET14b TEV EL vraE R | AAAGGATCCTTAACCGTTAGTAGCATCGAC | This study |

**References**

14. Meehl M, Herbert S, Götz F, Cheung A. Interaction of the GraRS Two-Component System with the VraFG ABC Transporter To Support Vancomycin-Intermediate Resistance in Staphylococcus aureus. AAC. 2007 Aug;51(8):2679–89.
