# Supplementary material for: The extracellular loop of the membrane permease VraG interacts with GraS to sense cationic antimicrobial peptides in Staphylococcus aureus
Source: PLoS Pathog. 2021 Mar 1;17(3):e1009338. doi: 10.1371/journal.ppat.1009338 (PMC7951975; doi:10.1371/journal.ppat.1009338)
Supplement: S2 Table — (DOCX) [file ppat.1009338.s009.docx]

| Strains and plasmids | Features | Reference |
| --- | --- | --- |
| IM08B pMAD::*graS* | IM08B with pMAD vector harboring flanking sequences (about 1,000 bp) upstream and downstream of *graS* gene | This study |
| IM08B pMAD::*vraG* | IM08B with pMAD vector harboring flanking sequences (about 1,000 bp) upstream and downstream of *vraG* gene | This study |
| IM08B pMAD::EL of *vraG* | IM08B with pMAD vector harboring flanking sequences (about 1,000 bp) upstream and downstream of the extracellular loop of *vraG* | This study |
| IM08B pMAD::*vraG* mutant 1 | IM08B with pMAD vector harboring flanking sequences (about 1,000 bp) upstream and downstream to mutate target residues in the VraG gene (K327A, K331A and K343A) | This study |
| IM08B pMAD::*vraG* mutant 2 | IM08B with pMAD vector harboring flanking sequences (about 1,000 bp) upstream and downstream to mutate target residues in the VraG gene (K351A, K357A, K360A, K367A and K369A) | This study |
| IM08B pMAD::*vraG* mutant 3 | IM08B with pMAD vector harboring flanking sequences (about 1,000 bp) upstream and downstream to mutate target residues in the VraG gene (K380A and K388A) | This study |
| IM08B pMAD::*vraG* mutant 4 | IM08B with pMAD vector harboring flanking sequences (about 1,000 bp) upstream and downstream to mutate target residues of the VraG gene (K402A, K406A, K408A, K409A, K412A, K418A and K419A) | This study |
| IM08B pMAD::*vraG* mutant 5 | IM08B with pMAD vector harboring flanking sequences (about 1,000 bp) upstream and downstream to mutate target residues in the VraG gene (K425A, K427A and K432A) | This study |
| IM08B pMAD::*vraG* mutant 6 | IM08B with pMAD vector harboring flanking sequences (about 1,000 bp) upstream and downstream to mutate target residues of the VraG gene (K451A, K458A, K461 and K463A) | This study |
| IM08B pMAD::*vraG* mutant 7 | IM08B with pMAD vector harboring flanking sequences (about 1,000 bp) upstream and downstream to mutate target residues in the VraG gene (K474A, K476A, K477A, K485A, K486A, K488A, and K491) | This study |
| IM08B pMAD::*vraG* K380A | IM08B with pMAD vector harboring flanking sequences (about 1,000 bp) upstream and downstream to mutate the target residue in the VraG gene (K380A) | This study |
| IM08B pMAD::*vraG* K388A | IM08B with pMAD vector harboring flanking sequences (about 1,000 bp) upstream and downstream to mutate the target residue in the VraG gene (K388A) | This study |
| IM08B  pMAD::*graS c*omplement | IM08B with pMAD vector harboring flanking sequences (about 1,000 bp) upstream and downstream of *graS* + native *graS* | This study |
| IM08B  pMAD::*vraG c*omplement | IM08B with pMAD vector harboring flanking sequences (about 1,000 bp) upstream and downstream of *vraG* + native *vraG∆* | This study |
| JE2 with pMAD::∆*graS* | JE2 with plasmid for mutation | This study |
| JE2 with pMAD::∆*vraG* | JE2 with plasmid for mutation | This study |
| JE2 with pMAD::∆EL of *vraG* | JE2 with plasmid for mutation | This study |
| JE2 with pMAD::*vraG* mutant 1 | JE2 with plasmid for mutation | This study |
| JE2 with pMAD::*vraG* mutant 2 | JE2 with plasmid for mutation | This study |
| JE2 with pMAD::*vraG* mutant 3 | JE2 with plasmid for mutation | This study |
| JE2 with pMAD::*vraG* mutant 4 | JE2 with plasmid for mutation | This study |
| JE2 with pMAD::*vraG* mutant 5 | JE2 with plasmid for mutation | This study |
| JE2 with pMAD::*vraG* mutant 6 | JE2 with plasmid for mutation | This study |
| JE2 with pMAD::*vraG* mutant 7 | JE2 with plasmid for mutation | This study |
| JE2 with pMAD::*vraG* K380A | JE2 with plasmid for mutation | This study |
| JE2 with pMAD::*vraG* K388A | JE2 with plasmid for mutation | This study |
| JE2 Δ*graS* with  pMAD::*graS* complement | JE2 Δ*graS* mutant with pMAD::*graS* to introduce complementation | This study |
| JE2 Δ*vraG* with  pMAD::*vraG* complement | JE2 Δ*vraG* mutant with pMAD::*vraG* to introduce complementation | This study |
| JE2 *vraG* K380A  pMAD::*vraG* complement | JE2 *vraG* K380A mutant with pMAD::*vraG* to introduce complementation | This study |
| IM08B  pALC1484::*mprF* promoter | IM08B with pALC1484 harboring *mprF* promoter | This study |
| IM08B  pALC1484::*dltA* promoter | IM08B with pALC1484 harboring *dltA* promoter | This study |
| IM08B  pMAD::*vraG* HA | IM08B with pMAD flanking sequences (about 1,000 bp) upstream and downstream of vraG with HA tag | This study |
| IM08B  pMAD::EL *vraG* HA | IM08B with pMAD flanking sequences (about 1,000 bp) upstream and downstream of EL vraG with HA tag | This study |
| IM08B  pMAD::*vraG* mutant 3 HA | IM08B with pMAD flanking sequences (about 1,000 bp) upstream and downstream of vraG mutant 3 with HA tag | This study |
| IM08B  pMAD::*vraG* K380A HA | IM08B with pMAD flanking sequences (about 1,000 bp) upstream and downstream of vraG K380A with HA tag | This study |
| IM08B  pMAD::*vraG* K388A HA | IM08B with pMAD flanking sequences (about 1,000 bp) upstream and downstream of vraG K388A with HA tag | This study |
| JE2 with pMAD::*vraG* HA | JE2 with plasmid for mutation | This study |
| JE2 ΔEL *vraG* with pMAD::EL *vraG* HA | JE2 with plasmid for mutation | This study |
| JE2 *vraG* Mutant 3 with pMAD::*vraG* mutant 3 HA | JE2 with plasmid for mutation | This study |
| JE2 *vraG* K380A with pMAD::*vraG* K380A HA | JE2 with plasmid for mutation | This study |
| JE2 *vraG* K388A with pMAD::*vraG* K388A HA | JE2 with plasmid for mutation | This study |
