# Supplementary material for: The extracellular loop of the membrane permease VraG interacts with GraS to sense cationic antimicrobial peptides in Staphylococcus aureus
Source: PLoS Pathog. 2021 Mar 1;17(3):e1009338. doi: 10.1371/journal.ppat.1009338 (PMC7951975; doi:10.1371/journal.ppat.1009338)
Supplement: S3 Table — This table shows the summary of MICs of PMB, mprF expression, cytochrome c binding and 2 hr. LL-37 susceptibility assays. Expression of mprF is relative comparison. For cytochrome c binding, the parent JE2 is set at 100%. For LL-37 assay, survival without LL-37 is set at 100%. The symbols indicate the following: ↑↑ / ↑ / ↓ / ↓↓ (high / moderate / low / considerably low expression. (DOCX) [file ppat.1009338.s010.docx]

| **Strains** | **MICs of PMB (μg/ml)** | ***mprF* expression** | **cytochrome c binding** | **Survival against LL-37** |
| --- | --- | --- | --- | --- |
| JE2 | 128 | ↑ | - (control) | ↑ |
| JE2 Δ*vraG* | 8 | ↓ | ↑↑ | ↓ |
| JE2 ΔEL of *vraG* | 16 | ↑↑ | ↓↓ | ↑↑ |
| JE2 *vraG* mutant 3 | 32 | ↑↑ | ↓ | ↑↑ |
| JE2 *vraG* K380A | 32 | ↑↑ | ↓ | ↑↑ |
| JE2 Δ*graS* | 4 | ↓ | ↑↑ | ↓ |
